# Genetic determinants of risk in pulmonary arterial hypertension: international genome-wide association studies and meta-analysis

**DOI:** 10.1016/S2213-2600(18)30409-0

**Published:** 2019-03

**Authors:** Christopher J Rhodes, Ken Batai, Marta Bleda, Matthias Haimel, Laura Southgate, Marine Germain, Michael W Pauciulo, Charaka Hadinnapola, Jurjan Aman, Barbara Girerd, Amit Arora, Jo Knight, Ken B Hanscombe, Jason H Karnes, Marika Kaakinen, Henning Gall, Anna Ulrich, Lars Harbaum, Inês Cebola, Jorge Ferrer, Katie Lutz, Emilia M Swietlik, Ferhaan Ahmad, Philippe Amouyel, Stephen L Archer, Rahul Argula, Eric D Austin, David Badesch, Sahil Bakshi, Christopher Barnett, Raymond Benza, Nitin Bhatt, Harm J Bogaard, Charles D Burger, Murali Chakinala, Colin Church, John G Coghlan, Robin Condliffe, Paul A Corris, Cesare Danesino, Stéphanie Debette, C Gregory Elliott, Jean Elwing, Melanie Eyries, Terry Fortin, Andre Franke, Robert P Frantz, Adaani Frost, Joe G N Garcia, Stefano Ghio, Hossein-Ardeschir Ghofrani, J Simon R Gibbs, John Harley, Hua He, Nicholas S Hill, Russel Hirsch, Arjan C Houweling, Luke S Howard, Dunbar Ivy, David G Kiely, James Klinger, Gabor Kovacs, Tim Lahm, Matthias Laudes, Rajiv D Machado, Robert V MacKenzie Ross, Keith Marsolo, Lisa J Martin, Shahin Moledina, David Montani, Steven D Nathan, Michael Newnham, Andrea Olschewski, Horst Olschewski, Ronald J Oudiz, Willem H Ouwehand, Andrew J Peacock, Joanna Pepke-Zaba, Zia Rehman, Ivan Robbins, Dan M Roden, Erika B Rosenzweig, Ghulam Saydain, Laura Scelsi, Robert Schilz, Werner Seeger, Christian M Shaffer, Robert W Simms, Marc Simon, Olivier Sitbon, Jay Suntharalingam, Haiyang Tang, Alexander Y Tchourbanov, Thenappan Thenappan, Fernando Torres, Mark R Toshner, Carmen M Treacy, Anton Vonk Noordegraaf, Quinten Waisfisz, Anna K Walsworth, Robert E Walter, John Wharton, R James White, Jeffrey Wilt, Stephen J Wort, Delphine Yung, Allan Lawrie, Marc Humbert, Florent Soubrier, David-Alexandre Trégouët, Inga Prokopenko, Richard Kittles, Stefan Gräf, William C Nichols, Richard C Trembath, Ankit A Desai, Nicholas W Morrell, Martin R Wilkins, UK NIHR BioResource Rare Diseases Consortium, UK PAH Cohort Study Consortium, US PAH Biobank Consortium

**Affiliations:** a Department of Medicine, Imperial College London, London, UK; b National Heart and Lung Institute, Imperial College London, London, UK; c Department of Surgery, University of Arizona, Tucson, AZ, USA; d Pharmacy Practice and Science, University of Arizona, Tucson, AZ, USA; e Department of Medicine, University of Arizona, Tucson, AZ, USA; f Department of Medicine, University of Cambridge, Cambridge, UK; g Department of Haematology, University of Cambridge, Cambridge, UK; h Molecular and Clinical Sciences Research Institute, St George's University of London, London, UK; i Sorbonne Universités, UPMC, INSERM, Paris, France; j Human Genetics, Cincinnati, OH, USA; k CAGE, Cincinnati, OH, USA; l Biomedical Informatics, Cincinnati, OH, USA; m Cincinnati Children's Hospital Medical Center, Cincinnati, OH, USA; n University Paris-Sud, Université Paris-Saclay, Le Kremlin-Bicêtre, Paris, France; o Data Science Institute, Lancaster University, Lancaster, UK; p Genetics and Molecular Medicine, King's College London, London, UK; q University of Giessen and Marburg Lung Center, Giessen, Germany; r University of Iowa, Iowa City, IA, USA; s University of Lille, Lille, France; t Queen's University, Kingston, ON, Canada; u Medical University of South Carolina, Charleston, SC, USA; v Vanderbilt University, Nashville, TN, USA; w University of Colorado Denver, Denver, CO, USA; x Baylor Research Institute, Plano, TX, USA; y Medstar Health, Washington, DC, USA; z Allegheny-Singer Research Institute, Pittsburgh, PA, USA; aa Ohio State University, Columbus, OH, USA; ab VU University Medical Center, Amsterdam, Netherlands; ac Mayo Clinic Florida, Jacksonville, FL, USA; ad Washington University, St Louis, MO, USA; ae Golden Jubilee National Hospital, Glasgow, UK; af Royal Free Hospital, London, UK; ag Royal Hallamshire Hospital, Sheffield, UK; ah University of Newcastle, Newcastle, UK; ai University of Pavia, Pavia, Italy; aj University of Bordeaux, Bordeaux, France; ak Intermountain Medical Center, Murray, UT, USA; al University of Cincinnati, Cincinnati OH, USA; am Duke University Medical Center, Durham, NC, USA; an University of Kiel, Kiel, Germany; ao Mayo Clinic, Rochester, MN, USA; ap Houston Methodist Research Institute, Houston, TX, USA; aq Fondazione IRCCS Policlinico San Matteo, Pavia, Italy; ar Tufts-New England Medical Center, Boston, MA, USA; as Health Sciences Center, University of Colorado, Aurora, CO, USA; at Rhode Island Hospital, Providence, RI, USA; au Ludwig Boltzmann Institute for Lung Vascular Research, Graz, Austria; av Indiana University, Indianapolis, IN, USA; aw University of Lincoln, Lincoln, UK; ax Royal United Hospitals Bath NHS Foundation Trust, Bath, UK; ay Great Ormond Street Hospital, London, UK; az Inova Heart and Vascular Institute, Falls Church, VA, USA; ba Harbor-UCLA Medical Center, Torrance, CA, USA; bb Papworth Hospital, Papworth, UK; bc East Carolina University, Greenville, NC, USA; bd Vanderbilt University School of Medicine, Nashville, TN, USA; be Columbia University, New York, NY, USA; bf Wayne State University, Detroit, MI, USA; bg University Hospital of Cleveland, Cleveland, OH, USA; bh Boston University School of Medicine, Boston, MA, USA; bi University of Pittsburgh, Pittsburgh, PA, USA; bj Ambry Genetics, Aliso Viejo, CA, USA; bk University of Minnesota, Minneapolis, MN, USA; bl University of Texas Southwestern Medical Center, Dallas, TX, USA; bm Louisiana State University Health, Shreveport, LA, USA; bn University of Rochester Medical Center, Rochester, NY, USA; bo Spectrum Health Hospitals, Grand Rapids, MI, USA; bp Seattle Children's Hospital, Seattle, WA, USA; bq University of Sheffield, Sheffield, UK; br City of Hope, Duarte, CA, USA

## Abstract

**Background:**

Rare genetic variants cause pulmonary arterial hypertension, but the contribution of common genetic variation to disease risk and natural history is poorly characterised. We tested for genome-wide association for pulmonary arterial hypertension in large international cohorts and assessed the contribution of associated regions to outcomes.

**Methods:**

We did two separate genome-wide association studies (GWAS) and a meta-analysis of pulmonary arterial hypertension. These GWAS used data from four international case-control studies across 11 744 individuals with European ancestry (including 2085 patients). One GWAS used genotypes from 5895 whole-genome sequences and the other GWAS used genotyping array data from an additional 5849 individuals. Cross-validation of loci reaching genome-wide significance was sought by meta-analysis. Conditional analysis corrected for the most significant variants at each locus was used to resolve signals for multiple associations. We functionally annotated associated variants and tested associations with duration of survival. All-cause mortality was the primary endpoint in survival analyses.

**Findings:**

A locus near *SOX17* (rs10103692, odds ratio 1·80 [95% CI 1·55–2·08], p=5·13 × 10^–15^) and a second locus in *HLA-DPA1* and *HLA-DPB1* (collectively referred to as *HLA-DPA1/DPB1* here; rs2856830, 1·56 [1·42–1·71], p=7·65 × 10^–20^) within the class II MHC region were associated with pulmonary arterial hypertension. The *SOX17* locus had two independent signals associated with pulmonary arterial hypertension (rs13266183, 1·36 [1·25–1·48], p=1·69 × 10^–12^; and rs10103692). Functional and epigenomic data indicate that the risk variants near *SOX17* alter gene regulation via an enhancer active in endothelial cells. Pulmonary arterial hypertension risk variants determined haplotype-specific enhancer activity, and CRISPR-mediated inhibition of the enhancer reduced *SOX17* expression. The *HLA-DPA1/DPB1* rs2856830 genotype was strongly associated with survival. Median survival from diagnosis in patients with pulmonary arterial hypertension with the C/C homozygous genotype was double (13·50 years [95% CI 12·07 to >13·50]) that of those with the T/T genotype (6·97 years [6·02–8·05]), despite similar baseline disease severity.

**Interpretation:**

This is the first study to report that common genetic variation at loci in an enhancer near *SOX17* and in *HLA-DPA1/DPB1* is associated with pulmonary arterial hypertension. Impairment of SOX17 function might be more common in pulmonary arterial hypertension than suggested by rare mutations in *SOX17*. Further studies are needed to confirm the association between HLA typing or rs2856830 genotyping and survival, and to determine whether HLA typing or rs2856830 genotyping improves risk stratification in clinical practice or trials.

**Funding:**

UK NIHR, BHF, UK MRC, Dinosaur Trust, NIH/NHLBI, ERS, EMBO, Wellcome Trust, EU, AHA, ACClinPharm, Netherlands CVRI, Dutch Heart Foundation, Dutch Federation of UMC, Netherlands OHRD and RNAS, German DFG, German BMBF, APH Paris, INSERM, Université Paris-Sud, and French ANR.

**Research in context**

**Evidence before this study**

We searched PubMed for research articles published in English before Aug 23, 2018, with search terms including “pulmonary arterial hypertension”, “genetics”, and “GWAS”. Rare genetic variation, primarily in genes associated with transforming growth factor-β family members, including *BMPR2*, but also in the transcription factor *SOX17*, is known to cause pulmonary arterial hypertension. However, little is known about the contribution of common variation to this disorder. Additionally, both pulmonary vascular endothelial dysfunction and altered immune and inflammatory signalling are observed in pulmonary arterial hypertension, but the underlying genetic mechanisms are poorly characterised.

**Added value of this study**

To our knowledge, this is the largest genetic analysis of pulmonary arterial hypertension to date, comprising more than 2000 patients with pulmonary arterial hypertension from four international cohorts. We identified one locus near *SOX17* and another in *HLA-DPA1* and *HLA-DPB1* that reached genome-wide significance, and we cross-validated these loci by meta-analysis. The *SOX17* locus includes two independent signals, both of which identify enhancer regions that specifically regulate the expression of *SOX17*, which is essential for pulmonary vascular development. Allelic variation at *HLA-DPB1* is associated with clinical outcomes, specifically survival, with more than two-thirds of patients harbouring genotypes associated with the poorest outcomes.

**Implications of all the available evidence**

Common variation near *SOX17* is a risk factor for pulmonary arterial hypertension and dysregulation of SOX17 might be more common in pulmonary arterial hypertension than the occurrence of rare variants suggests. Further studies are needed to define whether HLA typing or rs2856830 genotyping improves risk stratification in clinical practice and in clinical trials.

## Introduction

Pulmonary arterial hypertension refers to an uncommon but devastating disorder characterised by obliterative pulmonary vascular remodelling, leading to a progressive increase in pulmonary vascular resistance and right heart failure. Annual mortality for idiopathic and heritable pulmonary arterial hypertension remains around 10%, despite the use of modern therapies.^1^, ^2^ The high mortality partly reflects the limited effect of licensed treatments on the underlying pulmonary vascular pathology, which includes vascular smooth muscle and fibroblast hyperplasia, endothelial cell proliferation, and inflammation.^3^ Substantial variation between patients in their response to available treatments highlights underlying and inadequately characterised heterogeneity in the causes of pulmonary arterial hypertension.

Recent gene sequencing studies^4^, ^5^, ^6^ have revealed rare mutations in several genes, including *BMPR2*, genes encoding potassium channels, and most recently the transcription factor *SOX17*. Rare genetic variation is associated with both the risk of developing pulmonary arterial hypertension and survival, and it is found in up to 25% of patients with pulmonary arterial hypertension. In the majority of patients with pulmonary arterial hypertension, the extent of genetic contribution, including that attributable to common variation, remains largely unknown.^7^, ^8^ Therefore, we aimed to test for genome-wide association for pulmonary arterial hypertension in large international cohorts and assess the contribution of associated regions to patient outcomes (panel). This is the first report of the associations found at *SOX17* and *HLA-DPA1* and *HLA-DPB1* (collectively referred to as *HLA-DPA1/DPB1* in this Article).

Panel **Key terms**

**Genome-wide association study**

A genetic analysis approach, typically using millions of common variants (eg, single-nucleotide polymorphisms [SNPs]) covering the genome, to test whether an allele of a genetic variant is associated with a disease or trait, or the levels of a continuous trait of interest.

**Common variant**

An SNP for which the frequency of the less frequent allele is at least 5% in a given population. Typically, a common variant has subtle biological effects, as opposed to rare variants (also known as mutations), which can cause diseases or extreme phenotypes.

**Genetic locus**

A position on the genome defined by the chromosome number and the genetic distance in centimorgans (cM) or physical distance in base pairs (bps) on the chromosome. A locus can refer to a gene or a non-coding region of varying length (eg, from one hundred to millions of bps).

**Credible set**

A set of variants that is statistically likely (eg, with 99% probability) to contain the causal variant for the disease or trait of interest at a genetic locus.

## Methods

### Pulmonary arterial hypertension cohorts and genotyping

We did two genome-wide association studies and a meta-analysis on pulmonary arterial hypertension. Pulmonary arterial hypertension was defined by haemodynamic criteria according to international guidelines.^2^ Unrelated individuals with idiopathic, heritable, or anorexigen-associated pulmonary arterial hypertension were included. Individuals with evidence of other known causes of pulmonary arterial hypertension were excluded; therefore, no patients were known to have pulmonary arterial hypertension associated with clinically diagnosable autoimmune diseases (appendix pp 2–3). All enrolled individuals provided written informed consent from their respective institutions or were included as anonymous controls under the DNA databank at Vanderbilt University, BioVU, opt-out policy (appendix p 2).

Given the rarity of pulmonary arterial hypertension, four studies were used for the analyses. In the UK National Institute for Health Research BioResource (NIHRBR) Rare Diseases study, whole-genome sequencing (Illumina, San Diego, CA, USA; mean depth of around 35×; appendix p 2) was done in 5895 individuals of European descent, each with a rare disorder from 16 categories or their unaffected relatives, and 847 had pulmonary arterial hypertension (appendix p 4). The concept of this study was to sequence patients with rare diseases to identify genetic influences on the pathogenesis of one rare disorder using the other rare diseases as controls, assuming that distinct rare diseases are highly unlikely to share common genetic mechanisms. This assumption was tested by repeating analyses excluding each major control group (appendix p 6).

Three studies used genome-wide genotyping arrays: the US National Biological Sample and Data Repository for Pulmonary Arterial Hypertension (also known as PAH Biobank [PAHB]) study^9^ included 694 individuals with pulmonary arterial hypertension and 1560 controls ascertained for a large pharmacogenomic study at Vanderbilt University (Nashville, TN, USA); the Pulmonary Hypertension Allele-Associated Risk (PHAAR) study^7^ included 269 individuals with pulmonary arterial hypertension and 1068 population-based controls from France; and the British Heart Foundation Pulmonary Arterial Hypertension (BHFPAH) study consisted of 275 individuals with pulmonary arterial hypertension and 1983 population-based controls from several European countries (appendix p 12). All genotyping studies were imputed (appendix p 12), and single-nucleotide polymorphisms (SNPs) with good imputation quality (r^2^≥0·3) were taken forward for testing. Individuals from NIHRBR, PHAAR, and BHFPAH were tested for relatedness to prevent inclusion of the same or related individuals across studies. Other quality-control steps are detailed in the appendix (pp 4, 5, 12).

### Association analyses

We used logistic regression to test single-marker variants for genetic association with a diagnosis of pulmonary arterial hypertension assuming a log-additive genetic model and adjusting for sex, read length chemistry (NIHRBR only), and population structure using the first four (NIHRBR and PHAAR), three (PAHB), or ten (BHFPAH) principal components. We calculated the genomic inflation factor, which was verified to be between 1 and 1·05 for each study.

We used two independent sets for discovery: whole-genome sequencing data from NIHRBR (n=5895, including 847 pulmonary arterial hypertension cases); and meta-analysis of genotyping studies PAHB, PHAAR, and BHFPAH (n=5849, including 1238 pulmonary arterial hypertension cases). We cross-validated findings and confirmed loci in a meta-analysis of all four studies using the inverse variance-weighted fixed-effects approach (which maximises power for discovery studies^10^), implemented in the GWAMA software tool.^11^ Random-effects meta-analysis was subsequently applied to estimate generalisability of the results to different populations.^10^ We did a conditional analysis including the lead variant in each locus as a covariate to test for independent distinct signals reaching p<5×10^–8^.

We used LDlink to assess linkage disequilibrium of variants in all European populations from the 1000 Genomes Project. Credible sets of variants considered 99% likely to include the functional causal variants were calculated by summing ranked posterior probabilities (appendix p 6).

### Annotation and functional assessment of the locus near SOX17

The locus near *SOX17* was assessed against publicly available functional annotation datasets (including ENCODE, Factorbook Motifs, and Blueprint). The locus was investigated using CRISPR-mediated repression in human pulmonary artery endothelial cells (hPAECs; PromoCell GmbH, Heidelberg, Germany) by transduction with a lentivirus containing a plasmid encoding the nuclease-deficient Cas9 (dCas9) fused to the repressor KRAB and a 20 bp guide RNA (appendix pp 6–7). Cells were harvested following blasticidin selection, and the expression of *SOX17* as well as neighbouring *MRPL15* and *TMEM68* was assessed by quantitative PCR.

In-vitro enhancer activity of the loci and variants near *SOX17* was investigated using a luciferase reporter assay. Specifically, genomic DNA was isolated from endothelial progenitor cells (also known as blood outgrowth endothelial cells) derived from a patient with pulmonary arterial hypertension who was heterozygous for the lead SNP at *SOX17* and used to clone 100 bp putative enhancer regions containing the *SOX17* pulmonary arterial hypertension variants. The cloned products were inserted into a luciferase reporter plasmid, which was subsequently used for transformation of stable bacteria. Picking various bacterial colonies allowed for isolation of luciferase reporter plasmids containing genomic DNA inserts differing only by the allele of the SNP of interest. Reporter plasmids were transfected into hPAECs by electroporation, and luciferase activity was measured to quantify the enhancer function of the inserts with the relevant haplotype.

### Statistical analysis

Loci associated with pulmonary arterial hypertension were tested for associations with clinical variables (appendix pp 17–18). All-cause mortality was the primary endpoint in survival analyses using Kaplan-Meier estimates and Cox regression in the survival package in R, version 3.3.0.^12^ Survival was calculated from diagnosis to date of death or censoring (Oct 31, 2016, for NIHRBR; Aug 1, 2017, for PAHB; Sept 27, 2017, for PHAAR; Oct 12, 2017, for BHFPAH), with left truncation using date of genetic consent, and patients were censored at lung or heart-and-lung transplantation. Age and sex were covariates to correct for their association with prognosis.^2^ NIHRBR and PAHB were analysed separately, and PHAAR and BHFPAH were combined before analysis because of their smaller sample sizes. Cox regression results from these three analyses were then meta-analysed using the default random-effects model restricted maximum-likelihood estimator method implemented in the metafor package in R, version 3.3.0.^13^ All cohorts were combined for Kaplan-Meier analysis. We did sensitivity analyses excluding pathogenic *BMPR2* variant carriers, all pathogenic rare variant carriers, and patients diagnosed in previous decades who might have been exposed to different treatment regimens.

HLA alleles and amino acids totalling 1873 features were determined by imputation from genotyped and high-quality imputed variants in the HLA region using the SNP2HLA software and the type 1 diabetes genetics consortium reference database.^14^ HLA alleles and amino acids were tested for association with the novel lead variants or case-control status by χ^2^ test with false discovery rate correction.

### Role of the funding source

The funders of the study had no role in study design, data collection, data analysis, data interpretation, or writing of the report. CJR, KB, MB, MHa, LSo, MG, MWP, CH, AA, KBH, JHK, MK, AU, LH, JA, EMS, and SGr had access to raw data for analyses. The corresponding authors had full access to all the data in the study and had final responsibility for the decision to submit for publication.

## Results

**Figure 1:**
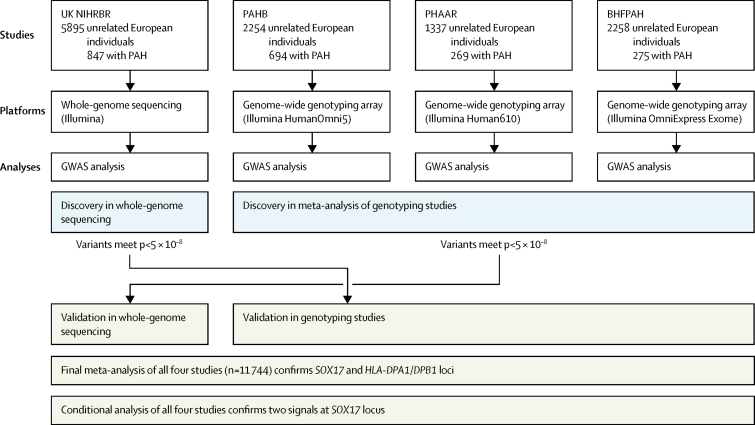
Study design *HLA-DPA1* and *HLA-DPB1* are collectively referred to as *HLA-DPA1/DPB1* in this Article. BHFPAH=British Heart Foundation Pulmonary Arterial Hypertension study. GWAS=genome-wide association study. NIHRBR=National Institute for Health Research BioResource study. PAH=pulmonary arterial hypertension. PAHB=PAH Biobank study. PHAAR=Pulmonary Hypertension Allele-Associated Risk study.

In two separate GWAS discovery analyses (figure 1), we identified two loci associated with pulmonary arterial hypertension reaching genome-wide significance (p<5 × 10^–8^; table 1; appendix p 23). One locus was 100–200 kb upstream of the transcription factor *SOX17*. A second locus was within *HLA-DPA1*/*DPB1*, which encodes the MHC class II DP α and β chains.

**Table 1:** Novel loci associated with pulmonary arterial hypertension in sequenced and genotyped cohorts

	**Chromosome and position, hg19:effect/non-effect alleles**	**Effect allele frequency in non-Finnish Europeans in gnomAD**	**Effect allele frequency in NIHRBR controls**	**UK NIHRBR whole-genome sequencing study (847 cases *vs* 5048 controls)**		**Effect allele frequency in genotyping controls**	**Meta-analysis of genotyping studies PAHB, PHAAR, and BHFPAH (1238 cases *vs* 4611 controls)**		**Meta-analysis of all cohorts (2085 cases, 9659 controls, n_eff_=6648)**	
				Odds ratio (95% CI)	p value		Odds ratio (95% CI)	p value	Odds ratio (95% CI)	Meta-analysis p value
**Lead SNPs**										
*HLA-DPA1/DPB1*, rs2856830	6:33041734:C/T	0·12	0·12	1·71 (1·48–1·96)	4·41 × 10^−14^*	0·13	1·44 (1·26–1·64)	5·35 × 10^−8^	1·56 (1·42–1·71)	7·65 × 10^−20^*
*SOX17*, signal 1 rs13266183	8:55267612:C/T	0·73	0·73	1·44 (1·26–1·64)	4·44 × 10^−8^*	0·74	1·31 (1·17–1·46)	4·1 × 10^−6^	1·36 (1·25–1·48)	1·69 × 10^−12^*
*SOX17,* signal 2 rs10103692	8:55258127:G/A	0·90	0·90	1·85 (1·47–2·31)	9·51 × 10^−8^	0·91	1·76 (1·45–2·14)	9·84 × 10^−9^*	1·80 (1·55–2·08)	5·13 × 10^−15^*
**Other SNPs in same loci**										
*HLA-DPB1* missense SNP, rs1042140	6:33048640:G/A	0·23	0·23	1·38 (1·22–1·55)	9·21 × 10^−8^	0·23	1·44 (1·29–1·61)	9·73 × 10^−11^*	1·41 (1·30–1·53)	7·13 × 10^−17^*
*SOX17,* genotyping lead SNP, rs28576721†	8:55265980:T/C	0·91	0·92	1·55 (1·23–1·95)	1·57 × 10^−4^	0·92	1·96 (1·57–2·43)	1·54 × 10^−9^*	1·75 (1·50–2·05)	3·07 × 10^−12^*

Odds ratios are for association between effect allele and pulmonary arterial hypertension. gnomAD is the Genome Aggregation Database, which provides information including allele frequencies in different populations. *HLA-DPA1* and *HLA-DPB1* are collectively referred to as *HLA-DPA1/DPB1* in this Article. BHFPAH=British Heart Foundation Pulmonary Arterial Hypertension study. n_eff_=number of individuals that would make up an equally powered study with a 1:1 case:control ratio (appendix p 2). NIHRBR=National Institute for Health Research BioResource study. PAH=pulmonary arterial hypertension. PAHB=PAH Biobank study. PHAAR=Pulmonary Hypertension Allele-Associated Risk study. SNP=single-nucleotide polymorphism.

* Significant.

† This is the most significant *SOX17* SNP after combining the three genotyping studies (not including NIHRBR) and forms part of signal 2.

**Figure 2:**
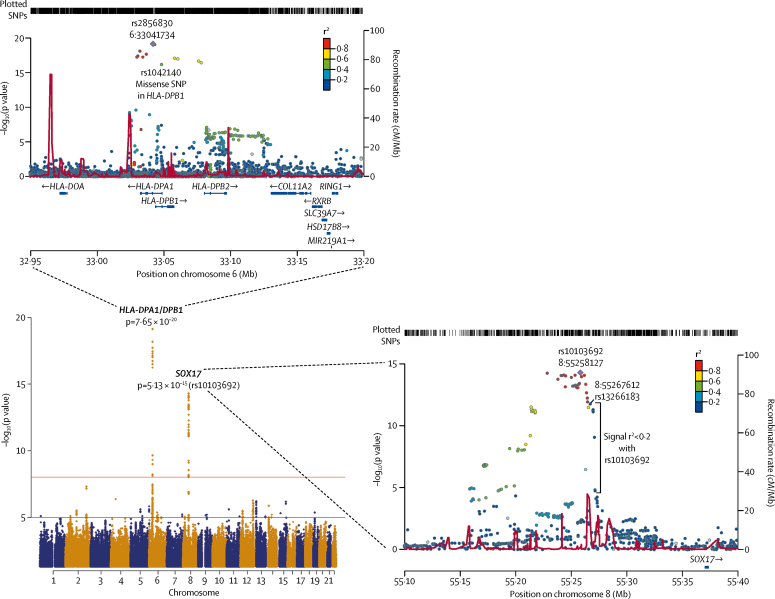
A meta-analysis of all cohorts and regional plots of novel loci The regional plots indicate variant location at the *HLA-DPA1* and *HLA-DPB1* (collectively referred to as *HLA-DPA1/DPB1* in this Article) locus and linkage disequilibrium structure at *SOX17* locus. At the *SOX17* locus, several variants associated with pulmonary arterial hypertension are in very weak or no linkage disequilibrium (r^2^<0·2) with the lead single-nucleotide polymorphism (SNP), rs10103692. We refer to these variants as *SOX17* signal 1 and the most significant variant, rs13266183, is indicated. The variants coloured as in linkage disequilibrium with rs10103692 comprise signal 2.

Both the *SOX17* and *HLA-DPA1*/*DPB1* loci reached genome-wide significance in the discovery analyses; our cross-validation strategy confirmed that the same alleles were more frequent in pulmonary arterial hypertension than in other disease or population controls in both analyses (table 1). The genome-wide meta-analysis of all four studies confirmed their associations with pulmonary arterial hypertension (rs2856830, odds ratio 1·56 [95% CI 1·42–1·71], p=7·65 × 10^–20^ for *HLA-DPA1*/*DPB1*; rs10103692, 1·80 [1·55–2·08], p=5·13 × 10^–15^ for *SOX17*; table 1; figure 2; appendix p 13). We detected no further loci at genome-wide significance. Allele frequencies in the different control groups were similar between studies and to non-Finnish Europeans in the public database gnomAD (table 1).

The conditional analysis confirmed that the *HLA-DPA1*/*DPB1* locus contained a single signal of association, but showed that the *SOX17* locus was composed of two independent signals; signal 1 is 100–103 kb upstream of *SOX17* (p_conditional_=9·82 × 10^–9^) and signal 2 is 106–200 kb upstream of *SOX17* (p_conditional_=4·16 × 10^–11^; figure 2; appendix p 15). The lead SNPs for the two signals in the *SOX17* locus were rs13266183 (for signal 1, odds ratio 1·36 [1·25–1·48], p=1·69 × 10^–12^) and rs10103692 (signal 2). A Bayesian credible set analysis to narrow the variants in these loci to those 99% likely to be causal (appendix p 15) showed that the *HLA-DPA1*/*DPB1* locus included nine SNPs (all p<9·1 × 10^–18^), *SOX17* signal 1 included four SNPs 100–103 kb upstream of *SOX17* (all p<3·3 × 10^–8^), and *SOX17* signal 2 included 31 SNPs 106–142 kb upstream of *SOX17* (all p<5·7 × 10^–10^).

Previous studies have reported the association of variants near *CBLN2*, and *PDE1A* and *DNAJC10* with pulmonary arterial hypertension.^7^, ^8^ These common variant signals showed no association with pulmonary arterial hypertension in the combined NIHRBR, PAHB, and BHFPAH cohorts (p=0·17 for *CBLN2* and p=0·24 for *PDE1A* and *DNAJC10*; appendix p 14). Sensitivity analyses excluding pathogenic *BMPR2* variant carriers, all pathogenic rare variant carriers, and controls from different disease groups yielded similar results to the main analyses (appendix p 9).

**Figure 3:**
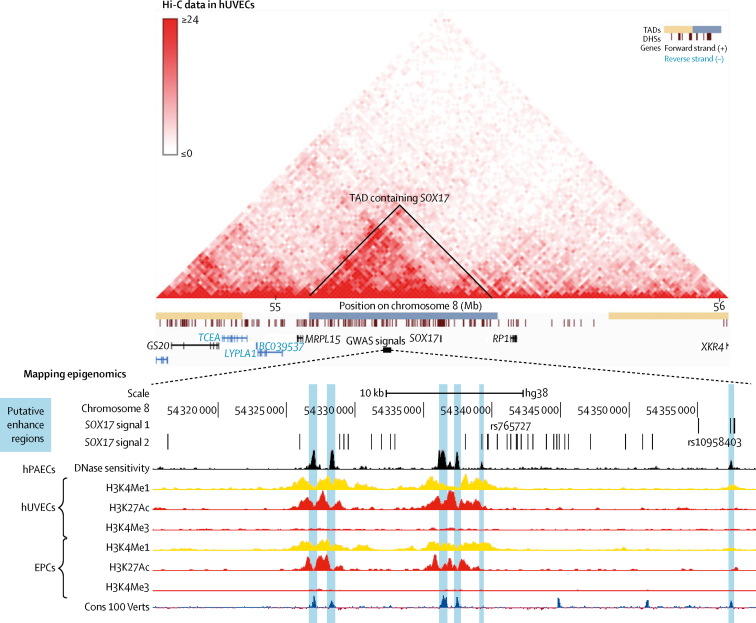
In-silico analysis of *SOX17* locus Hi-C data from human umbilical vein endothelial cells (hUVECs) indicate regions of DNA found in close proximity in the three-dimensional structure. The genomic region containing the significant variants identified by the genome-wide association study (GWAS) analysis is indicated by a black box, overlapping a topologically associated domain (TAD) indicated in blue, which contains only *SOX17.* Mapping of *SOX17* locus variants associated with pulmonary arterial hypertension with public epigenomic data is underneath Hi-C data. The credible set indicates positions of variants 99% likely to contain the causal variants. Auxiliary hidden Markov models, which summarise epigenomic data to predict the functional status of genomic regions in different tissues or cells, are shown. Epigenomic data in endothelial cells including hUVECs, human pulmonary artery endothelial cells (hPAECs), and endothelial progenitor cells (EPCs), indicate areas likely to contain active regulatory regions and promoters. Markers include histone H3 lysine 4 monomethylation (H3K4Me1; often found in enhancers) and trimethylation (H3K4Me3; strongly observed in promoters) and H3 lysine 27 acetylation (H3K27Ac; often found in active regulatory regions). The blue vertical blocks indicate where epigenomic data suggest a putative enhancer region, some overlapped by variants associated with pulmonary arterial hypertension. These regions were cloned for the luciferase reporter experiments (figure 4B). DHSs=DNase I hypersenstivity sites.

To search for evidence of regulatory elements in relevant tissues at *SOX17* signal 1 and signal 2, we examined publicly available epigenomic data (including histone modifications; figure 3; appendix p 24). We identified several putative enhancer elements active in both lung tissue and endothelial cells (figure 3). One of these elements (around hg19-chr8:55·270 Mb) contains a cluster of three of four credible variants from *SOX17* signal 1 (figure 3). Another (around hg19-chr8:55·252 Mb) contains one credible variant from *SOX17* signal 2. Of these variants, rs10958403 in signal 1 and rs765727 in signal 2 overlap a DNase I hypersensitivity signal, which indicates accessible chromatin (allowing binding of transcription factors), detected in hPAECs (figure 3).

**Figure 4:**
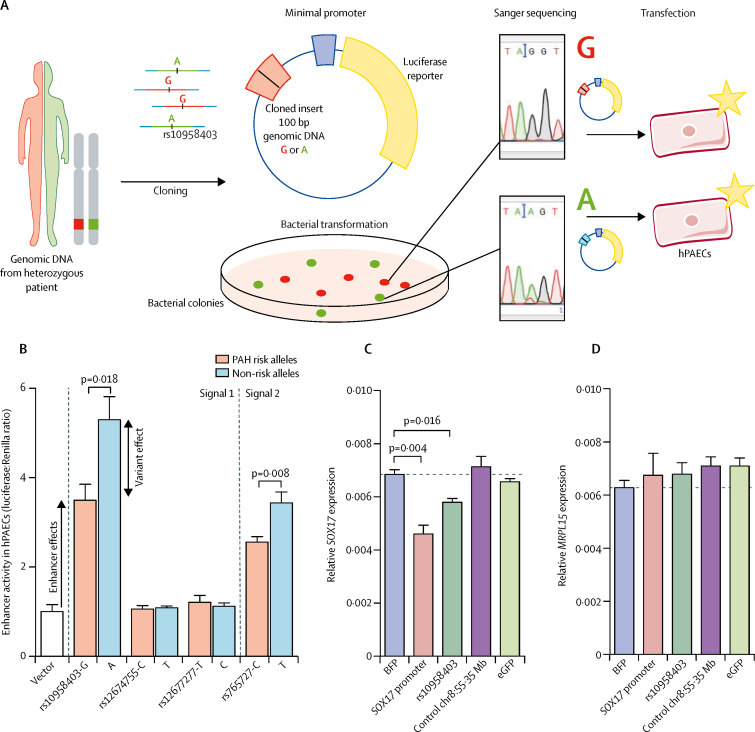
In-vitro analysis of *SOX17* locus (A) Process for haplotype-specific reporter construct derivation. 100 bp genomic DNA inserts containing *SOX17* single-nucleotide polymorphisms (SNPs) are isolated from endothelial progenitor cells derived from a patient with pulmonary arterial hypertension (PAH) heterozygous for the *SOX17* SNPs. Colonies of transformed bacteria can be sequenced to determine alleles present in the product. Transfection of luciferase reporter constructs containing inserts into human pulmonary artery endothelial cells (hPAECs) allows for determination of luciferase activity. (B) Luciferase reporter assay results. Luciferase:Renilla ratios relative to the empty vector demonstrate haplotype-dependent enhancement of promoter activity. Enhancer effects were tested by one-way analysis of variance followed by Dunnett's post-hoc tests: rs10958403-G/A and rs765727-C/T were both p<0·0001 significant versus empty vector; variant effects of these two SNPs were tested by *t* test. The mean (SEM; error bars) of five experiments is shown. (C) Relative expression of *SOX17:ACTB* in hPAECs on CRISPR-mediated repression of the near *SOX17* genome-wide association study (GWAS) locus. The mean (SEM; error bars) of four measurements in a representative experiment is shown. Three further experiments showed consistent results. Blue fluorescent protein (BFP), enhanced green fluorescent protein (eGFP), and control, which refers to a region between the enhancer region and the *SOX17* gene that is negative for regulatory markers, are used as negative controls. The *SOX17* promoter was targeted as a positive control of repression. Significance shown versus BFP by Dunnett's post-hoc analysis. (D) Relative expression of *MRPL15:ACTB* in hPAECs on CRISPR-mediated repression of the GWAS locus.

To study the effects of the pulmonary arterial hypertension risk variants on the putative enhancers defined by the epigenomic signals, we developed reporter constructs containing 100 bp of the regions containing either the risk allele or non-risk alleles at each of the four SNPs using genomic DNA from a patient heterozygous for both *SOX17* signals. A haplotype-specific reporter assay in hPAECs confirmed that the regions containing either rs10958403 or rs765727 exhibited enhancer activity (between threefold and sixfold induction of luciferase:Renilla ratio, p<0·0001), whereas constructs containing rs12674755 or rs12677277 had no effect compared with the empty vector control (figure 4). We also observed haplotype-specific activity with the active constructs, which differed only by the alleles at pulmonary arterial hypertension-associated risk variants rs10958403 or rs765727 (both p<0·05; figure 4).

DNA folding patterns determined by Hi-C data from lung tissue and endothelial cells (human umbilical vein endothelial cells [figure 3] and human microvascular endothelial cells [data not shown]) indicate that the *SOX17* pulmonary arterial hypertension locus resides in a defined topologically associated domain in which the only gene found, and thus likely target of any regulatory elements in this region, is *SOX17.* CRISPR-mediated inhibition of the *SOX17* signal 1 region in hPAECs resulted in selective downregulation of *SOX17* expression but not the expression of neighbouring genes *MRPL15* and *TMEM68*, suggesting that the enhancers in this locus specifically regulate *SOX17* (figure 4; appendix p 26).

**Figure 5:**
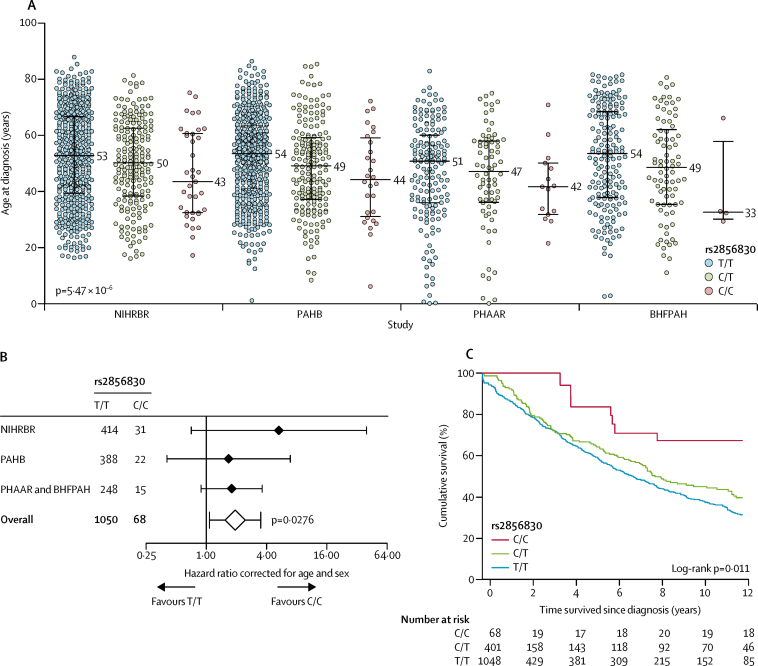
Clinical impact of *HLA-DPB1* rs2856830 (A) Age at diagnosis by genotype in four cohorts of patients with pulmonary arterial hypertension (PAH). Bars indicate medians (IQRs); numbers given are median values in subgroups. The p value shown is from a linear regression model correcting for cohort differences. (B) Forest plot showing hazard ratios for the rs2856830 6:33041734 T/T versus C/C genotypes, corrected for age and sex in Cox regression survival analyses in each cohort, individually and with meta-analysis results. Error bars indicate 95% CIs. (C) Kaplan-Meier survival plot in patients with pulmonary arterial hypertension divided into groups on the basis of the genotype of *HLA-DPA1* and *HLA-DPB1* single-nucleotide polymorphism (SNP) rs2856830 in all cohorts. Numbers at risk indicates numbers at risk in each time period, which increases as truncated patients are recruited into the study after diagnosis and decreases as patient follow-up ends. Significance from the log rank test is given. BHFPAH=British Heart Foundation Pulmonary Arterial Hypertension study. NIHRBR=National Institute for Health Research BioResource study. PAHB=PAH Biobank study. PHAAR=Pulmonary Hypertension Allele-Associated Risk study.

We investigated whether the *HLA-DPA1*/*DPB1* and *SOX17* variants affect clinical outcomes in pulmonary arterial hypertension, specifically all-cause mortality. The *HLA-DPA1*/*DPB1* rs2856830 genotype, but not the *SOX17* locus, was strongly associated with survival (figure 5). Median survival from diagnosis in patients with pulmonary arterial hypertension with the C/C homozygous genotype was double (13·50 years [95% CI 12·07 to >13·50]) that of those with the T/T genotype (6·97 years [6·02–8·05]). Cox regression survival analyses showed that the rs2856830 T/T genotype conferred an increased annual risk of death in pulmonary arterial hypertension (hazard ratio [HR] 1·94 [95% CI 1·08–3·51]; figure 5).

Sensitivity analyses excluding pathogenic *BMPR2* variant carriers, all pathogenic rare variant carriers, and patients diagnosed in previous decades who might have been exposed to different treatment regimens gave results similar to the main analyses (appendix p 27).

We tested both loci for association with other clinical variables, including disease severity measures and comorbidities (appendix pp 17–18). The C allele at *HLA-DPA1*/*DPB1* lead SNP rs2856830 was associated with younger age at diagnosis (figure 5), with C/C homozygotes presenting a decade earlier than T/T homozygotes (appendix p 17). The rs2856830 genotype was not associated with vasoresponder status.

The *HLA-DPA1*/*DPB1* locus included a missense variant rs1042140 in *HLA-DPB1* reaching genome-wide significance (table 1) in partial linkage disequilibrium (r^2^=0·45 with lead rs2856830 in Europeans). The SNP rs1042140 determines a glutamic acid (Glu^69^) or a lysine at amino acid residue 69. To determine specific HLA alleles associated with the lead variant, rs2856830, we imputed HLA types from the genotype data. These types are represented by digit codes, where the first two digits represent related groups of similar alleles (eg, DPB1*02), and four digits represent specific proteins with distinct amino acid sequences (eg, DPB1*02:01). We found that the pulmonary arterial hypertension-enriched C allele of rs2856830 was associated with HLA-DPB1*02:01/02:02/16:01 (all p<1 × 10^–9^ after false discovery rate correction; table 2; appendix p 19), which all contain the Glu^69^ residue. The most numerous DPB1*02:01 and DPB1*04:01 alleles were associated with survival in patients with pulmonary arterial hypertension (HR 0·70 [95% CI 0·49–1·00] for DPB1*02:01 and 1·33 [1·04–1·70] for DPB1*04:01; appendix p 21).

**Table 2:** Associations of *HLA-DPB1* alleles with the lead SNP rs285683

	**Amino acid residues in *HLA-DPB1* alleles**																			**Frequencies by GWAS SNP rs2856830**			**q after FDR correction**
	8	9	11	33	35	36	55	56	57	65	69	76	84	85	86	87	96	178	194	T/T	T/C	C/C	
DPB1^*^02:01†	L	F	G	E	F	V†	D†	E†	E	I	E†	M	G	G	P	M	R	L	R	244/8870 (3%)	1182/2682 (44%)	211/234 (90%)	<5 × 10^−247^
DPB1^*^02:02†	L	F	G	E	L	V†	E	A	E	I	E†	M	G	G	P	M	..	..	..	2/8870 (<1%)	93/2682 (3%)	16/234 (7%)	2·77 × 10^−87^
DPB1^*^16:01†	L	F	G	E	F	V†	D†	E†	E	I	E†	M	D	E	A	V	..	..	..	4/8870 (<1%)	64/2682 (2%)	5/234 (2%)	7·08 × 10^−41^
DPB1^*^03:01‡	V	Y	L	E	F	V†	D†	E†	D	L	K‡	V	D	E	A	V	K	L	R	1094/8870 (12%)	152/2682 (6%)	0/234 (0%)	5·50 × 10^−23^
DPB1^*^04:01‡	L	F	G	E	F	A‡	A‡	A‡	E	I	K‡	M	G	G	P	M	R	L	R	4251/8870 (48%)	697/2682 (26%)	1/234 (<1%)	2·40 × 10^−138^
DPB1^*^04:02‡	L	F	G	E	F	V†	D†	E†	E	I	K‡	M	G	G	P	M	R	M	R	1153/8870 (13%)	166/2682 (6%)	0/234 (0%)	2·08 × 10^−23^
DPB1^*^01:01‡	V	Y	G	E	Y	A‡	A‡	A‡	E	I	K‡	V	D	E	A	V	K	L	Q	611/8870 (7%)	99/2682 (4%)	0/234 (0%)	1·17 × 10^−8^

Data are n/N (%) unless otherwise stated. FDR=false discovery rate. GWAS=genome-wide association study. SNP=single-nucleotide polymorphism.

† Alleles and residues depleted in pulmonary arterial hypertension cases.

‡ Alleles and residues enriched in pulmonary arterial hypertension cases.

The risk alleles at both signals within the *SOX17* locus are common (risk allele frequencies are 74% for rs13266183-C and 92% for rs9298503-C), such that 1230 (59%) of 2085 patients with pulmonary arterial hypertension were homozygous for the risk allele at both *SOX17* SNPs, compared with 4443 (46%) of 9659 controls.

The alleles at *HLA-DPB1* associated with the poorest outcomes are also common (risk allele frequency of 86% for rs2856830-T), such that 1432 (69%) of 2085 patients with pulmonary arterial hypertension had the T/T genotype associated with the poorest outcomes and 1975 (95%) of 2085 patients had at least one T allele.

## Discussion

Through a meta-analysis of 11 744 individuals, we have established loci at an enhancer upstream of *SOX17* and at *HLA-DPA1/DPB1* associated with pulmonary arterial hypertension disease risk. Common genetic variants in the enhancer region of *SOX17* are biologically plausible candidates for susceptibility to pulmonary vascular disease. Polymorphic variation at the *HLA-DPA1/DPB1* locus is strongly associated with both the age at diagnosis and prognosis in pulmonary arterial hypertension.

Both in-silico and experimental analyses of the common variants upstream of the *SOX17* gene suggest that they affect susceptibility to pulmonary arterial hypertension through regulation of *SOX17* expression. We provided direct evidence that inhibition of *SOX17* signal 1 reduced *SOX17* expression, and luciferase activity experiments showed a functional variant in both signals. Combined with the in-silico data, it seems highly likely that signal 2 would also be targeting *SOX17* because no other gene is present in the topologically associated domain and promoter capture HiC data show that this area associates with the *SOX17* promoter. We have recently reported^4^ enrichment and familial segregation in pulmonary arterial hypertension of causal rare deleterious variation in *SOX17*, implicating this gene in the pathogenesis of pulmonary arterial hypertension. SOX17 is involved in the development of the endoderm,^15^, ^16^, ^17^ vascular endothelium, haemopoietic cells,^18^ and cardiomyocytes.^19^, ^20^ SOX17 also determines the endothelial fate of CD34 progenitor cells de-differentiated from fibroblasts.^21^ Deletion in the mouse leads to abnormal pulmonary vascular development, poor distal lung perfusion and biventricular hypertrophy.^22^ SOX17 is a pro-angiogenic transcription factor and interacts with well established endothelial molecular mediators;^23^, ^24^ reduction of SOX17 in endothelial cells through Notch activation (which is associated with BMPR2 signalling^25^) restricts angiogenesis.^23^ Conversely, vascular endothelial growth factor (VEGF) upregulates SOX17 and, as part of a positive-feedback loop, SOX17 promotes expression of VEGF receptor 2.^24^

We report that *HLA-DPB1* alleles are associated with pulmonary arterial hypertension and have a pivotal role in determining disease progression. The beneficial effect of the C/C genotype at rs2856830 on survival is clinically significant, extending average survival from about 7 to about 14 years, despite no apparent difference in baseline disease severity by standard clinical measures, including haemodynamics and exercise capacity. Patients with the C allele at rs2856830 presented at a significantly younger age than those with the T allele, but the association of the *HLA-DPB1* SNP with survival remains significant after correction for both age and sex. The somewhat conflicting observation that the C/C genotype is associated with earlier, more frequent presentation but improved survival compared with the C/T or T/T genotypes perhaps suggests that there could be two different mechanisms involved; one that affects initial disease pathogenesis and another that alters the adaptation to the established disease state. A parallel in pulmonary arterial hypertension is the paradox of female prevalence contrasted with poorer outcomes for male patients,^2^ although the mechanisms for this still remain unclear. Further evaluation of this survival association in independent datasets would help to define how clinical HLA typing or rs2856830 genotyping could improve risk stratification in clinical practice and in clinical trials, in which over-representation of the C/C genotype in one treatment group could significantly affect outcomes.

The mechanism of rs2856830 involvement in pulmonary arterial hypertension is probably through its association with specific *HLA-DPB1* alleles. Class II (HLA-DRB1, HLA-DQB1, and HLA-DPB1) antigen-presenting proteins have crucial roles in the adaptive immune response.^26^, ^27^ The *HLA-DPB1* alleles associated with rs2856830 (HLA-DPB1*02:01/02:02/16:01) in the current study have also previously been linked to susceptibility to hard metal lung diseases, such as berylliosis.^28^, ^29^ A number of individual amino acid residues in the peptide-binding pockets of the HLA-DPB1 molecule affect its function and T-cell recognition, either by changing peptide antigen binding or the conformation of the peptide-binding groove.^30^ HLA-DPB1*02:01/ 02:02/16:01 all contain a glutamate at position 69 and a valine at position 36 that reduce the risk of clinical deterioration. These same residues are essential for T-cell activation and cytokine production in berylliosis.^31^, ^32^ The potential role of this modification in antigen binding, autoimmune response, and vascular damage in pulmonary arterial hypertension demands further investigation.

To examine whether the associations observed were driven by *trans* effects of known rare pathogenic variants in pulmonary arterial hypertension, we did sensitivity analyses that demonstrated that the associations were independent of *BMPR2* and other rare pathogenic variants. Although the sequencing and array platforms used in this study might perform differently across the genome, the signals detected for each platform remained strong.

This study has some limitations. The majority of patients studied were prevalent cases, and the association with survival is only marginally significant and based on a relatively small sample size, from multiple studies with different ascertainment criteria. Thus, confirmation of the survival analysis in an independent sample of patients recruited at diagnosis would greatly increase confidence in this finding. Some variants displayed heterogeneity of effects between studies, which is most likely due to the limited sample size in the smaller genotyping studies. Variants in *CBLN2* and other loci previously associated with pulmonary arterial hypertension^7^, ^8^ were not replicated by this study, suggesting that these previous findings were either false positives or only relevant to the specific subpopulations studied.

We have shown in a rare disorder that common variation can drive significant clinical differences in presentation and outcomes. Furthermore, a common non-coding variant can regulate expression of a gene linked by rare, deleterious mutations to the same disease. *HLA-DPB1*, and wider immune regulatory pathways, should be considered a priority for patient stratification and investigation of new treatments in pulmonary arterial hypertension. *SOX17* is a key endothelial regulator and its dysfunction in pulmonary arterial hypertension might be more common than suggested by the occurrence of rare pathogenic variants in heritable cases.

Comprehensive affiliations listed in the appendix
