# Supplementary material for: Genetic determinants of risk in pulmonary arterial hypertension: international genome-wide association studies and meta-analysis
Source: Lancet Respir Med. 2019 Mar;7(3):227–38. doi: 10.1016/S2213-2600(18)30409-0 (PMC6391516; doi:10.1016/S2213-2600(18)30409-0)
Supplement: Supplementary appendix [file mmc1.pdf]

THE LANCET
Respiratory Medicine

Supplementary appendix

This appendix formed part of the original submission and has been peer reviewed. We post it as supplied by the authors.

Supplement to: Rhodes CJ, Batai K, Bleda M, et al, on behalf of the UK NIHR BioResource Rare Diseases Consortium, the UK PAH Cohort Study Consortium, and the US PAH Biobank Consortium. Genetic determinants of risk in pulmonary arterial hypertension: international genome-wide association studies and meta-analysis. *Lancet Respir Med* 2018; published online Dec 5. http://dx.doi.org/10.1016/S2213-2600(18)30409-0.

Supplementary Appendix for

Genetic determinants of risk and survival in pulmonary arterial hypertension

Page 2: Supplementary Methods

Page 9: Supplementary Results

Page 10: Supplementary References

Page 12: Supplementary Tables

Page 23: Supplementary Figures

Page 29: Legends for Supplementary Tables and Figures

Page 31: Full authorship and affiliations details

Page 33: NIHR BioResource Collaborators list

Page 38: PAH Biobank Collaborators lists

Authors: **Christopher J. Rhodes, Ph.D.*, Ken Batai, Ph.D.*, Marta Bleda, Ph.D.*, Matthias Haimel, B.Sc.*, Laura Southgate, Ph.D.*, Marine Germain, Ph.D.*, Michael W. Pauciulo, B.S., M.B.A.***, *et al.*

Corresponding authors contact details:
Ankit A. Desai, University of Arizona, Tucson, AZ, United States: adesai@shc.arizona.edu;
Nicholas W. Morrell, University of Cambridge, Cambridge, United Kingdom: nwm23@cam.ac.uk;
Martin R. Wilkins, Imperial College London, London, United Kingdom: m.wilkins@imperial.ac.uk.

Supplementary methods

Effective sample size (N_{eff}), which is the number of individuals which would make up an equally-powered study with a 1:1 case:control ratio, is defined as $4/[(1/N_{cases})+(1/N_{controls})]$). It is provided to allow more direct comparison of the power of each study to detect associations.

BMPR2 mutations were considered pathogenic if they had been classified as clearly or likely pathogenic variants[1].

Study Cohorts

UK National Institute of Health Research BioResource (NIHRBR) for Rare Diseases study – PAH was defined by right heart catheterization measurements including mean pulmonary artery pressure (mPAP) > 25 mmHg, pulmonary capillary wedge pressure (PCWP) < 15 mmHg, and pulmonary vascular resistance (PVR) > 3 Woods Units. Eligible cases were recruited from the UK National Pulmonary Hypertension Centres, as well as Université Sud Paris (France), the VU University Medical Center Amsterdam (The Netherlands), the Universities of Gießen and Marburg (Germany), and San Matteo Hospital, Pavia (Italy). Study recruitment was undertaken between 29 Jan 2003 and 4 Jan 2017, and patients were followed up to 24 Mar 2017. Patients with a family history or presence of known pathogenic mutations were included in the main analysis, but excluded from sub-analyses. Cases were excluded if they were not able to provide written informed consent or were diagnosed with other forms of PAH. One patient withdrew from the study. Controls consisted of patients with other rare diseases from the NIHRBR rare disease study (more details of final numbers below).

US National Biological Sample and Data Repository for Pulmonary Arterial Hypertension/PAH Biobank (PAHB) study - PAH was defined by right heart catheterization measurements including mPAP ≥ 25 mmHg, PCWP < 18 mmHg, and PVR > 2.5 Woods Units. Eligible cases were recruited from 29 pulmonary hypertension centers across the United States and enrolled as part of the National Biological Sample and Data Repository for Pulmonary Arterial Hypertension (PAH Biobank, www.pahbiobank.org) funded by the National Institutes of Health/National Heart Lung and Blood Institute (R24HL105333). PAH cases were recruited between October 3, 2012 to March 14, 2016. Controls were selected from the Vanderbilt Electronic Systems for Pharmacogenomic Assessment (VESPA) cohort[2-4] ascertained at Vanderbilt University (VU). The VESPA project used BioVU, VU's large DNA repository coupled to de-identified data from electronic health records (EHRs), to investigate the genetic component of individual response to medications for 28 pharmacogenomic phenotypes unrelated to PAH, including angiotensin-converting enzyme (ACE) inhibitor-induced cough, vancomycin-induced kidney dysfunction, heparin-induced thrombocytopenia, and others[2]. BioVU was approved by the Institutional Review Board at Vanderbilt University as described previously[5]. BioVU recruited using an opt-out model until January 2015, at which time an opt-in model was adopted. The complete VESPA project population includes 11,639 genotyped individuals from BioVU (84% Caucasian and 12% African American) with a median age of 61.6 years. Only samples of European descent that were genotyped on the Illumina® Omni5-Quad BeadChip array were included in the control population for this study (n=2,144)[6]. The controls include individuals with type 1 diabetes (n=251) and connective tissue diseases (n=56). 31 controls with a diagnosis of pulmonary hypertension were excluded. Combining cases with controls data (n=2,144), a total of 4,245 subjects were available for analysis.

Paris Pulmonary Hypertension Allele-Associated Risk cohort (PHAAR) study - Diagnosis with PAH was defined by hemodynamic measurement during right-heart catheterization for all cases identified

by the French PAH Network between 1 January, 2003, and 1 April, 2010. For all cases, PAH was defined as a mPAP >= 25 mmHg associated with normal PCWP. Cases with known pathogenic mutations in *BMPR2* or *ACVRL1* were excluded. Further details have been published[7]. The control group was composed of a random sample of 1,140 subjects who were free of any chronic disease from the 3C Study[8]. The 3C Study is a population-based prospective cohort with a 4-year follow-up carried out in three French cities: Bordeaux (southwest France), Montpellier (southeast France) and Dijon (central eastern France).

British Heart Foundation Pulmonary Arterial Hypertension GWAS (BHFPAH) study - The BHFPAH cohort comprised IPAH patients recruited from the Pulmonary Hypertension Division at University Hospital Giessen or from specialist PAH centres in the UK, namely Royal Hallamshire Hospital Pulmonary Vascular Unit, Northern Pulmonary Vascular Unit – Freeman Hospital, Papworth Hospital NHS Foundation Trust, National Pulmonary Hypertension Service – Hammersmith Hospital, Royal Brompton and Harefield NHS Foundation Trust, and the Scottish Pulmonary Vascular Unit. Patients were recruited between 3 Dec 1998 and 1 Dec 2011 and all provided written informed consent to participate in the study. The BHFPAH control cohort was population based, comprising of individuals ascertained through the Wellcome Trust Case Control Consortium, UK or recruited as part of the Food Chain Plus (FoCus) cohort[9], Germany. Individuals in the Focus cohort with BMI>30 were excluded. All IPAH cases and UK controls were genotyped at King's College London on an Illumina HiScan system, whilst genotype data for the German controls were generated by Popgen[10].

All patients in all cohorts studied were consented using either NIHRBR study consent forms (UK Research Ethics Committee: 13/EE/0325), PAHB consent forms (R24HL105333), BHFPAH consent forms (08/H0802/32) or local tissue bank consent forms allowing genetic testing. The use of DNA from the PAHB controls for genetics studies has been approved by the Institutional Review Board at VU as described previously)[3,5].

Vasoresponders are defined as patients who showed a significant drop in pulmonary artery pressure in response to acute vasodilator exposure at cardiac catheterisation. These patients subsequently go on to show good clinical outcomes on calcium channel blocker vasodilator therapy[11].

The data collected within each study were collected according to standard SOPs determined at the initiation of each study and monitored by a central site for each study.

Whole-genome DNA sequencing in NIHRBR study

DNA extracted from venous blood underwent whole-genome sequencing using the Illumina TruSeq DNA PCR-Free Sample Preparation kit (Illumina Inc., San Diego, CA, USA) and Illumina HiSeq 2500 or HiSeq X sequencer, generating 100 - 150 bp reads with a minimum coverage of 15X for ~95% of the genome (mean coverage of 35X). Throughout the project the read length chemistries used were 100 bp (n=357), then 125 bp (n=3074), and finally 150bp (n=5586). Sequencing reads were pre-processed by Illumina with Isaac Aligner and Variant Caller (v2, Illumina Inc.) using human genome assembly GRCh37 as reference. Variants were normalised and merged into a multi-sample VCF file using the gvcf aggregation tool 'agg'. Samples with potential handling errors (n=3) were excluded.

Genotyping of PAHB patient cohort and controls

DNA extracted from venous blood of both cases and controls was genotyped using Illumina HumanOmni5 Bead Chip system (Illumina, San Diego, CA). Genomic DNA prepared at the PAH

Biobank was used according to manufacturer's manual protocol contained in Illumina's Infinium LCG Quad Assay Protocol Guide. XStain LCG BeadChip step of the protocol was performed on a Tecan Freedom Evo robot (Tecan, Switzerland) and completed BeadChips were scanned on an Illumina iScan system.

Sample QC in NIHRBR Study

Female sex was defined by the $\log_{10}$-transformed YX coverage ratio being less than the mean plus three times the standard deviation in self-reported females (-1.027). Seven samples where sex and self-reported gender differed were excluded. Gender data were not available for 15 controls, these controls were also excluded.

The ethnic origin of each participating individual was determined using a representative set of 35,114 MAF>0.3, independent autosomal SNPs with no missing calls present on Illumina genotyping arrays[1]. Briefly, principal components were calculated in 2,110 European, East and South Asian and African samples from the 1,000G project and used to assign ethnicities to all 9,110 samples in the NIHRBR. Only individuals of European ancestry, as assigned from a mixture of multivariate Gaussian models[1], were considered for the discovery analysis (n=7,196). 251 South Asian subjects, who represented the largest ethnic group after Europeans, were used for a trans-ethnic meta-analysis.

Average heterozygosity and missingness was calculated for each individual within ethnic groups in all variants with MAF>0.05. All individuals with either measure more than 3 times the interquartile range from the median were excluded from the analysis (n=92).

Relatedness was defined by pi-hat scores calculated between all pairs of individuals (minimum pi-hat score in relatives was 0.094) using the same SNPs used for ethnicity calculations and the algorithm implemented in PC-Relate (GENESIS package, as previously described[1]). Related individuals were grouped into family networks and the (i) most inter-connected and/or (ii) first-sequenced individuals in each family were removed sequentially until no further related individuals remained (n=1,209 excluded).

To define remaining population structure principal components were calculated in the European individuals included in the analysis using the same 35,114 variants described above.

In the UK discovery analysis, the final group consisted of 5895 individuals including 847 PAH patients, recruits to Genomics England Ltd (GEL, n=1,102, 21.8% of non-PAH), individuals with bleeding, thrombotic and platelet disorders (BPD, n=834, 16.5%), primary immune disorders (PID, n=802, 15.9%), retinal dystrophies/paediatric neurology and metabolic disease (SPEED, n=774, 15.3%), multiple primary tumours (MPMT, n=472, 9.4%), hypertrophic cardiomyopathy (HCM, n=202, 4.0%), intrahepatic cholestasis of pregnancy (ICP, n=184, 3.7%), steroid resistant nephrotic syndrome (SRNS, n=154, 3.1%), primary membranoproliferative glomerulonephritis (PMG, n=133, 2.6%), cerebral small vessel disease (CSVD, n=126, 2.5%), neuropathic pain disorder (NPD, n=117, 2.3%), stem cell and myeloid disorder (SMD, n=79, 1.6%), leber hereditary optic neuropathy (LHON, n=53, 1.1%), Ehlers-Danlos syndrome (EDS, n=11, 0.2%) and others (CNTRL, n=5, 0.1%), or their first degree relatives.

Sample QC in PAHB study

For PAH Biobank cases, raw data, call rates, and quality scores were visualized (Illumina GenomeStudio). Patient samples with low genotyping rate (<95%) and gender discordance between clinical data and genomic data (n=8) were excluded for final genotyping calling. SNPs with call frequency < 97%, cluster separation <0.45, AA R, AB R, and BB R mean ≤0.2, 10% GenCall score ≤0.3, Het Excess >0.2, A//B frequency >0.5, and AB T mean <0.15 or >0.85 were removed. For the controls**,** genotype calling was performed at Vanderbilt University (VU). A total of 2,101 PAH cases were successfully genotyped. Control genotype data were cleaned using the quality control pipeline developed by the eMERGE Genomics Working Group[6].

For each dataset, we excluded samples with (i) high degree of relatedness and (ii) race/ethnicity discordance between recorded clinical data and genomic data. Identity-By-Descent (IBD-pairwise) analysis was performed to identify potentially related or duplicated samples. After removing individuals self-identified as other than non-Hispanic European ancestry, case and control individuals' genomic ancestry was assessed using data from 1000 Genomes Project including individual of European, East and South Asian, and African ancestry through the use of principal component analysis. A total of 200,448 independent, common SNPs were included in the principal component analysis. Individuals who did not cluster with 1000 Genome Project European Populations were removed.

Imputation in PAHB study

The 1,740,956 successfully genotyped SNPs were included for imputation. Imputation was performed using Michigan Imputation Server[12]. After removing duplicated SNPs and SNPs with ambiguous alleles, and correcting strand, VCF files were uploaded to their server. The phasing of genotype data were done using Eagle version 2.3[13]. Imputation was performed using the Minimac3 algorithm and the Haplotype Reference Consortium panel[14]. After the imputation, there were a total of 39,148,816 SNPs. We excluded 31,421,226 SNPs due to insufficient quality control metrics and low frequency, including poor imputation quality (Rsq<0.3), MAF≤1%, and Hardy Weinberg Equilibrium P-value <0.00001. A total of 7,727,590 SNPs were included for statistical analysis.

QC in BHFPAH study

To ensure the replication cohort was independent from the samples used for GWAS discovery, the complete BHFPAH and NIHR-BR datasets were compared using identity-by-descent analysis in plink and related samples were removed from the BHFPAH cohort. Standard GWAS QC was performed on this subsample. Variant QC included selection of autosomes only, exclusion of duplicate and ambiguous variants, alignment to the forward strand (merged with 1000 Genomes), exclusion of variants with excess missingness (>0.05), exclusion of variants with significant differences in call rate between cases and controls, exclusion of rare variants (MAF<0.01), and exclusion of variants with significant HWE deviation (p<1x10-05 in controls). Sample QC included exclusion of heterozygosity outliers (>3SD), duplicated/ related individuals (IBD>0.1875), reported and genotyped sex mismatches, and population PCA outliers. The final cohort used for analysis comprised 275 cases (n=136 German, n=139 UK) and 1,983 controls (n=509 German FOCUS sample, n=1474 UK). Post QC, genotypes were imputed to 1000 Genomes phase 3 version 5 using the Michigan Imputation Server. Post imputation, SNPs were filtered on minor allele frequency (MAF>=0.01) and estimated

imputation accuracy (INFO/R2>=0.7). Logistic regression association analyses were run in PLINK with the first 10 principal components of the genotype data.

Sensitivity analyses

Sensitivity analyses excluding 1) cases with rare clearly pathogenic or likely pathogenic BMPR2 variants or 2) each of the disease control groups in the NIHRBR (rare diseases) and PAHB data were performed to evaluate the influence by these subgroups on the stability of observed effects.

Credible set analysis

We applied credible set fine-mapping[15] to dissect the potential causal variants driving the observed associations. The *P*-values from the meta-analysis results including all four studies were converted into Bayes' Factors (BF) following the notation of Fuchsberger *et al.*[16], and posterior probabilities for each BF were calculated by dividing each value by the sum of all BFs within +/- 200 kb region of the lead SNP. We further ranked the posterior probabilities from the largest to the smallest, and formed 99% credible sets by summing up the ranked posterior probabilities until the cumulative sum reached 0.99.

Effects of novel loci on clinical phenotypes

Associations between novel loci and clinical phenotypes, including age, sex, haemodynamics, PAH functional class and co-morbidities in PAH cases were tested using Kruskal Wallis ANOVA for continuous variables and Cochran-Armitage test (assuming an additive model) for categorical variables, or the linear-by-linear test for functional class. P-values corrected for false discovery rate (FDR)<0.05 were considered significant.

Assessment of the *SOX17* locus

Hi-C data from the Hi-C browser[17] were visualised at http://promoter.bx.psu.edu, accessed 1st May 2018. The *SOX17* locus was mapped using epigenetic modification data and functional annotations of the region using NIH Roadmap Epigenomics Mapping Consortium data including the auxiliary chromatin Multivariate Hidden Markov Model (chromHMM)[18,19] and specific chromatin modification information for relevant tissues from the EU-FP7 project Blueprint Epigenome study [http://www.blueprint-epigenome.eu/, accessed 17/07/17] and DNase hypersensitivity data in pulmonary artery endothelial cells from ENCODE[20], visualised using the UCSC Genome Browser (https://genome-euro.ucsc.edu/, accessed 17/07/17). Promoter capture Hi-C data were visualised using the chicp.org plotter[21]. We also queried the Genotype-Tissue Expression (GTEx) database (https://www.gtexportal.org, accessed 27/09/2017) for eQTL analysis.

Cell culture

Primary human pulmonary arterial endothelial cells were obtained from PromoCell (PromoCell GmbH, Heidelberg, Germany). Cells were seeded on fibronectin (Sigma Aldrich) and cultured in Endothelial Growth Medium 2 (PromoCell) with supplements as provided by the manufacturer. For experiments cell of passage 4-7 were used. Experiments were repeated with cells from different donors.

Repression of putative SOX17 enhancer region

A single CRISPR inhibition vector containing an expression cassette with a nuclease dead SpCas9 (dCas9) fused to the KRAB repressor domain, 2A peptide and blasticidin resistance, and an expression cassette for a Cas9 single guide RNA was used to repress targeted regions (lentiCRISPRi, lentiEF1a-KRAB-dCas9-2A-blast-(BB), Addgene#118154). Three different guide RNAs against the putative enhancer site (chr8:55269900-55270100, hg19) and against a non-coding region upstream of the *SOX17* transcription start site (chr8:55353563-55353583, hg19) were designed using Tefor CRISPOR tool (Version 4.3, May 2017) and cloned into the lentiCRISPRi vector. Validated guide RNA sequences against Enhanced Green Fluorescent Protein (EGFP) and blue fluorescent protein (BFP) were obtained from Addgene (https://www.addgene.org/crispr/reference/grna-sequence/; 118157, Lenti-(BB)-EF1a-KRAB-dCas9-P2A-BlastR BFP-guide1; 118158, Lenti-(BB)-EF1a-KRAB-dCas9-P2A-BlastR EGFP-guide1). Guide RNAs against *EGFP*, as well as a guide RNA against a non-coding region upstream of the *SOX17* transcription start site served as negative controls. A guide RNA against the transcription start site of *SOX17* served as positive control. After optimization, the guide RNA with the highest repression efficiency (based on *SOX17* expression) was chosen (Figure S9B) and used for subsequent experiments. The sequence of the guide RNAs is provided in Table S12.

Lentiviral particles were prepared by co-transfection of the CRISPRi lentiviral plasmids with lentiviral packaging plasmids pMDLg/pRRE (Addgene #12251), pRSV-Rev (Addgene #12253) and pMD2.G (Addgene #12259) packaging/envelope into 70% confluent human 293FT cells (Invitrogen) using PEIpro DNA transfection reagent (Polyplus Transfection, Illkirch France). Lentiviral supernatant was harvested 72 h after transfection, and concentrated with Lenti-X Concentrator (Clontech, Mountain View US) following manufacturer's instructions. Human pulmonary arterial endothelial cells were transduced with 10µl of concentrated lentivirus for 48h followed by a selection with 10µg/mL blasticidin (Gibco/Thermofisher Scientific, Hempstead UK) for 72h.

***SOX17* expression**

After 72h of blasticidin selection, cells were lysed and mRNA was extracted using Qiagen RNeasy kit (Qiagen, Manchester UK). Reverse transcriptase polymerase chain reaction (RT-PCR) was performed using Multiscribe Reverse Transciptase (Applied Biosystems/ThermoFisher Scientific), followed by quantitative PCR, using PowerSYBRgreen mastermix (Applied Biosystems/ThermoFisher Scientific). Relative expression of the gene of interest (GOI) was calculated as 2^(CT value [GOI] – CT value [*ACTB1*]), in which *ACTB1* served as housekeeping gene. In addition to *SOX17*, the relative expression of two neighbouring genes (*MRPL15* and *TMEM68*) was measured. The primer sequences used for qPCR were designed using PrimerBlast, and are provided in Table S12.

Haplotype-specific Luciferase assay

Genomic DNA (gDNA) was isolated from blood-outgrowth endothelial cells (BOECs) using a QiaAmp DNA Mini kit (Qiagen). BOECs were isolated and cultured as previously described[22]; for haplotype-specific reporter assays gDNA was isolated from a patient heterozygous for rs13266183. Primers were designed (http://nebuilder.neb.com/) to clone 100bp gDNA areas containing each of the SNPs in the credible set which overlapped the epigenomic data indicating likely enhancer function (Figure 2B). Primers were flanked by sequences compatible with KpnI restriction sites (Table S12). Inserts were obtained by Q5-based polymerase chain reactions (New England Biolabs, Ipswich, Massachusetts), and purified after gel electrophoresis. The purified product was cloned into the multiple cloning site of pGL4.23 firefly luciferase vector (Promega, Madison, Wisonsin), followed by transformation of the final vector and insert into stable Top10 E.coli. Plasmid DNA was extracted

from multiple bacterial colonies, and sent for Sanger sequencing to obtain plasmids with haplotype-specific inserts, i.e. A versus G at rs10958403. Human pulmonary artery endothelial cells (HPAECs) were transfected with either of these plasmids, using AMAXA electroporation (device: Nucleofector I, programme: M-03, kit: VPI-1001, Lonza, Basel, Switzerland). The empty pGL4.23 vector served as control, while a Renilla luciferase plasmid (Promega) was co-transfected to control for transfection efficiency. 24h after transfection cells were lysed, followed by measurement of firefly luciferase and Renilla luciferase (Dual Luciferase Reporter Assay, Promega). Data are presented as fold-induction of the luciferase/Renilla ratio compared to the empty vector.

Software

Bcftools (v1.2)[23], vcftools (v0.1.14)[24], plink (v1.90beta)[25], R[26] and RStudio[27] with associated packages (v3.3.0/v1.0.136, R Foundation for Statistical Computing, Vienna, Austria) IBM SPSS Statistics 23 (International Business Machines Corp, New York, NY) and Microsoft Excel 2010 (Microsoft, Redmond, WA) were used for analysis of data.

Supplementary Results

Definition of key variants and sensitivity analyses

Sets of variants most likely to be causal ('credible sets') at the two novel PAH loci comprised 9 variants at *HLA-DPA1/DPB1* and 4 and 31 at the two *SOX17* signals, respectively (Table S3).

The strength of association with the *HLA-DPA1/DPB1* and *SOX17* signals was not altered after exclusion of 161 (NIHRBR) and 54 (PAHB) cases with pathogenic[28] rare heterozygous variants in *BMPR2* (Table S4). Similar results were seen after removing patients with any monogenic cause of PAH, including *BMPR2* mutations (Table S4). In additional sensitivity analyses, removing other rare diseases from non-PAH controls did not change the strength of association with the top signals (Table S4).

Association between PAH and known expression quantitative trait loci (eQTL)

We queried the Genotype-Tissue Expression (GTEx) database (https://www.gtexportal.org, accessed 27/09/2017) to examine the potential downstream regulatory effects of the SNPs associated with PAH in different tissues but found none were associated with *Sox17* expression, and the *Sox17* eQTLs present in both GTEx and the discovery GWAS analyses (rs2375863, rs1123133, rs79519760 and rs142492583) were not associated with PAH ($p>0.5$ in all). For *HLA-DPB1* all eQTLs present in the GWAS analysis were in LD with rs1811359, which was also not associated with PAH ($p=0.9$).

Association between *HLA-DPB1* locus and outcomes in incident PAH

Survival analyses were repeated after removal of (i) cases with rare, pathogenic *BMPR2* mutations and (ii) any rare, pathogenic mutation in known PAH genes, with similar results (Figure S4). A sub-analysis within 192 NIHRBR/PAHB incident cases (enrolled within 6 months of diagnosis) showed, for rs2856830, a minor allele frequency, MAF=0.16 (versus MAF=0.12 in controls and MAF=0.20 in all cases) and a similar separation in survival curves by genotype (Figure S4E).

Analysis of 673 PAH patients diagnosed since 2010 with follow-up, who would have had access to all modern medications under the same treatment guidelines, showed a similar difference in survival by copies of the C allele of rs2856830, with estimated 5-year survival of 100% in C/C homozygotes [data not shown].

PAH locus at *HLA-DPA1/DPB1*

The lead variant rs2856830 is in weak LD with variants associated with vasculitis and systemic sclerosis, conditions which are themselves associated with PAH (rs9277341[29] [$r^2=0.31$] and rs3135021[30] [$r^2=0.33$]). The *HLA-DPA1/DPB1* locus was independently associated with PAH after conditioning on these variants ($p_{cond}=5.41\times10^{-9}$).

In a case-control analysis, *HLA-DPB1**02 (most commonly *02:01, $p=4.91\times10^{-11}$) was most enriched in PAH cases ($p=4.95\times10^{-12}$, Table S8). *HLA-DPB1**04 was less common in PAH, whilst *HLA-DRB1**15 and several HLA-B alleles including *B**35 (primarily *B**35:01) were enriched in PAH (all $p<0.05$, Figure S5). The residues that differ between the most common *HLA-DPB1**0201 (enriched) and *DPB1**0401 (depleted) alleles are at positions 36, 55, 56 and 69 (Table 2 and Table S9). These residues were also associated with rs2856830 genotype, diagnosis of PAH (versus controls) and survival in PAH patients (Table S9).

Supplementary References

1. Graf S, Haimel M, Bleda M, et al. Identification of rare sequence variation underlying heritable pulmonary arterial hypertension. Nat Commun 2018;9:1416.
2. Bowton E, Field JR, Wang S, et al. Biobanks and electronic medical records: enabling cost-effective research. Sci Transl Med 2014;6:234cm3.
3. Karnes JH, Cronin RM, Rollin J, et al. A genome-wide association study of heparin-induced thrombocytopenia using an electronic medical record. Thromb Haemost 2015;113:772-81.
4. Karnes JH, Shaffer CM, Bastarache L, et al. Comparison of HLA allelic imputation programs. PLoS One 2017;12:e0172444.
5. Roden DM, Pulley JM, Basford MA, et al. Development of a large-scale de-identified DNA biobank to enable personalized medicine. Clin Pharmacol Ther 2008;84:362-9.
6. Zuvich RL, Armstrong LL, Bielinski SJ, et al. Pitfalls of merging GWAS data: lessons learned in the eMERGE network and quality control procedures to maintain high data quality. Genet Epidemiol 2011;35:887-98.
7. Germain M, Eyries M, Montani D, et al. Genome-wide association analysis identifies a susceptibility locus for pulmonary arterial hypertension. Nat Genet 2013;45:518-21.
8. Group CS. Vascular factors and risk of dementia: design of the Three-City Study and baseline characteristics of the study population. Neuroepidemiology 2003;22:316-25.
9. Muller N, Schulte DM, Turk K, et al. IL-6 blockade by monoclonal antibodies inhibits apolipoprotein (a) expression and lipoprotein (a) synthesis in humans. J Lipid Res 2015;56:1034-42.
10. Nothlings U, Krawczak M. [PopGen. A population-based biobank with prospective follow-up of a control group]. Bundesgesundheitsblatt Gesundheitsforschung Gesundheitsschutz 2012;55:831-5.
11. Galie N, Humbert M, Vachiery JL, et al. 2015 ESC/ERS Guidelines for the diagnosis and treatment of pulmonary hypertension: The Joint Task Force for the Diagnosis and Treatment of Pulmonary Hypertension of the European Society of Cardiology (ESC) and the European Respiratory Society (ERS): Endorsed by: Association for European Paediatric and Congenital Cardiology (AEPC), International Society for Heart and Lung Transplantation (ISHLT). Eur Respir J 2015;46:903-75.
12. Das S, Forer L, Schonherr S, et al. Next-generation genotype imputation service and methods. Nat Genet 2016;48:1284-7.
13. Loh PR, Danecek P, Palamara PF, et al. Reference-based phasing using the Haplotype Reference Consortium panel. Nat Genet 2016;48:1443-8.
14. McCarthy S, Das S, Kretzschmar W, et al. A reference panel of 64,976 haplotypes for genotype imputation. Nat Genet 2016;48:1279-83.
15. Wellcome Trust Case Control C, Maller JB, McVean G, et al. Bayesian refinement of association signals for 14 loci in 3 common diseases. Nat Genet 2012;44:1294-301.
16. Fuchsberger C, Flannick J, Teslovich TM, et al. The genetic architecture of type 2 diabetes. Nature 2016;536:41-7.
17. Wang Y, Zhang B, Zhang L, et al. The 3D Genome Browser: a web-based browser for visualizing 3D genome organization and long-range chromatin interactions. bioRxiv 2017.
18. Ernst J, Kellis M. ChromHMM: automating chromatin-state discovery and characterization. Nat Methods 2012;9:215-6.
19. Roadmap Epigenomics C, Kundaje A, Meuleman W, et al. Integrative analysis of 111 reference human epigenomes. Nature 2015;518:317-30.
20. Thurman RE, Rynes E, Humbert R, et al. The accessible chromatin landscape of the human genome. Nature 2012;489:75-82.
21. Schofield EC, Carver T, Achuthan P, et al. CHiCP: a web-based tool for the integrative and interactive visualization of promoter capture Hi-C datasets. Bioinformatics 2016;32:2511-3.

22. Wojciak-Stothard B, Abdul-Salam VB, Lao KH, et al. Aberrant chloride intracellular channel 4 expression contributes to endothelial dysfunction in pulmonary arterial hypertension. Circulation 2014;129:1770-80.
23. Li H, Handsaker B, Wysoker A, et al. The Sequence Alignment/Map format and SAMtools. Bioinformatics 2009;25:2078-9.
24. Danecek P, Auton A, Abecasis G, et al. The variant call format and VCFtools. Bioinformatics 2011;27:2156-8.
25. Chang CC, Chow CC, Tellier LC, Vattikuti S, Purcell SM, Lee JJ. Second-generation PLINK: rising to the challenge of larger and richer datasets. Gigascience 2015;4:7.
26. R core team. R: A language and environment for statistical computing. R Foundation for Statistical Computing, Vienna, Austria https://www.R-project.org/. 2016.
27. RStudio Team. RStudio: Integrated Development for R. RStudio, Inc., Boston, MA URL http://www.rstudio.com/. 2015.
28. Gräf S, Haimel M, Bleda M, et al. Novel causative genes for heritable pulmonary arterial hypertension. bioRxiv 2017.
29. Xie G, Roshandel D, Sherva R, et al. Association of granulomatosis with polyangiitis (Wegener's) with HLA-DPB1*04 and SEMA6A gene variants: evidence from genome-wide analysis. Arthritis Rheum 2013;65:2457-68.
30. Gorlova O, Martin JE, Rueda B, et al. Identification of novel genetic markers associated with clinical phenotypes of systemic sclerosis through a genome-wide association strategy. PLoS Genet 2011;7:e1002178.

Supplementary tables

	UK NIHRBR	US PAHB	Paris PHAAR study	London BHFPAH study
Genotyping platform	Illumina WGS	Illumina HumanOmni5 Quad BeadChip	Illumina Human610-Quad DNA BeadChip	Illumina OmniExpress Exome BeadChip
Variant call rate	≥95%	≥95%	≥95%	≥95%
Hardy-Weinberg Equilibrium	$p>10^{-5}$	$p>10^{-5}$	$p>10^{-5}$	$p>10^{-5}$
Minor allele frequency (MAF)	>2%	>1%	>1%	>1%
Imputation quality	n/a	Rsq≥0.3	Rsq≥0.3	Rsq≥0.3
Filter on individuals included	Europeans, unrelated, gender matches sex, heterozygosity, missingness	Europeans, unrelated, gender matches sex, age≥18,	Europeans, unrelated, BMPR2 negative	Europeans, unrelated, gender matches sex, heterozygosity
Final N cases	847	694	269	275
Final N controls	5,048	1,560	1,068	1,983
Effective N	2,901	1,921	860	966
Sex: number female [%]	579 [68.4%]	539 [77.8%]	185 [73.4%]	184 [66.9%]
Age at diagnosis	50.8 [38.0 - 64.6]	52.4 [39.8 - 61.7]	48.9 [35.0 - 58.0]	51.3 [36.9 - 67.0]
Diagnosis [%]				
Idiopathic PAH	759 [89.6%]	612 [88.2%]	222 [82.5%]	246 [89.5%]
Heritable PAH	51 [6.0%]	54 [7.8%]	8 [3.0%]	27 [9.8%]
Drug-associated PAH	37 [4.4%]	28 [4%]	39 [14.5%]	2 [0.7%]
Years from diagnosis to sampling, median (interquartile range)	2.64 (0.79-6.60)	4.27 (1.40-8.51)	1.74 (0.07-5.75)	0 (0-1.93)
PAH rare mutations identified, (%)	178 (21.0%)	94 (13.5%)	0*	21 (7.6%)

Table S1 - Genotyping details and quality controls of studies. *patients with rare mutations in PAH genes were specifically excluded from recruitment to this study.

Variant	Chromosome and position, hg19 : Effect/ Non-effect alleles	Effect allele frequency in non-Finnish Europeans in gnomAD	Meta-analysis of all cohorts (n=2085 cases, 9659 controls, effective n=6648)		Random effects meta-analysis of all cohorts (n=2085 cases, 9659 controls, effective n=6648)		Measures of heterogeneity between cohorts			r2 if imputed		
Lead SNPs			Odds ratio (95% confidence intervals)	Meta-analysis P-value	Odds ratio (95% confidence intervals)	Meta-analysis P-value	I^2	Q	Sig. Q	PAHB	PHAAR	BHFPAH
HLA-DPA1/DPB1, rs2856830	6:33041734:C/T	0.12	1.56 (1.42 - 1.71)	$7.65x10^{-20}$	1.55 (1.40 - 1.71)	$2.21x10^{-17}$	0.088	3.29	1	0.99380	0.992	0.99729
SOX17, signal 1 rs13266183	8:55267612:C/T	0.73	1.36 (1.25 - 1.48)	$1.69x10^{-12}$	1.32 (1.13 - 1.54)	$4.87x10^{-4}$	0.667	9.01	1	0.99841	0.9567	0.97445
SOX17, signal 2 rs10103692	8:55258127:G/A	0.90	1.80 (1.55 - 2.08)	$5.13x10^{-15}$	1.80 (1.55 - 2.08)	$5.13x10^{-15}$	0	1.94	1	0.99950	0.9951	0.99478
Other SNPs												
HLA-DPB1 missense SNP, rs1042140	6:33048640:G/A	0.23	1.41 (1.30 - 1.53)	$7.13x10^{-17}$	1.41 (1.30 - 1.53)	$5.77x10^{-17}$	0	0.73	1	0.97427	0.8518	0.96113
SOX17, genotyping lead SNP rs28576721*	8:55265980:T/C	0.91	1.75 (1.50 - 2.05)	$3.07x10^{-12}$	1.78 (1.44 - 2.19)	$5.85x10^{-8}$	0.342	4.56	1	0.92054	0.5513	0.91886

Table S2 - Heterogeneity and imputation quality of lead SNPs across studies. I^2 - Heterogeneity index I2. Q; heterogeneity index, with calculated significance p-values (Sig. Q).

Variant	*CBLN2*: PHAAR GWAS SNP, rs2217560	*PDE1A|DNAJC10:* Japanese GWAS SNP 1, rs71427857	*PDE1A|DNAJC10:* Japanese GWAS SNP 2, rs13023449
Chromosome and position, hg19	18:70150939	2:183497840	2:183499313
Effect/ Non-effect alleles	G/A	T/G	C/T
**UK NIHRBR (n=847 cases v 5,048 controls)**			
Effect allele frequency in controls/cases	0.084 / 0.099	0.09 / 0.10	0.09 / 0.10
Odds ratio (95% confidence intervals)	1.19 (0.99 - 1.42)	1.10 (0.92 - 1.31)	1.10 (0.92 - 1.31)
Sig.	0.061	0.31	0.29
**US PAHB (n=694 cases v 1,560 controls)**			
Effect allele frequency in controls/cases	0.083 / 0.082	0.08 / 0.09	0.08 / 0.09
Odds ratio (95% confidence intervals)	1.05(0.82 - 1.34)	1.00 (0.80 - 1.27)	1.00 (0.80 - 1.27)
Sig.	0.74	0.98	0.98
r2 if imputed	genotyped	0.99955	genotyped
**Paris PHAAR study (n=269 cases v 1,068 controls)**			
Effect allele frequency in controls/cases		0.10 / 0.10	0.10 / 0.10
Odds ratio (95% confidence intervals)	*Study first reported CBLN2 SNP*	0.99 (0.70 - 1.41)	1.0 (0.70 - 1.42)
Sig.		0.97	0.99
r2 if imputed	genotyped	0.9942	0.9987
**London BHFPAH study (n=275 cases v 1,983 controls)**			
Effect allele frequency in controls/cases	0.083 / 0.083	0.08 / 0.10	0.08 / 0.10
Odds ratio (95% confidence intervals)	0.97 (0.70 - 1.35)	1.17 (0.86 - 1.60)	1.17 (0.86 - 1.60)
Sig.	0.87	0.308	0.308
r2 if imputed	0.99254	0.99956	genotyped
**Meta-analysis of UK NIHRBR, US PAHB and London BHFPAH (n=1,816 vs 8,591) and Paris PHAAR for PDE1A/DNAJC10 (n=2,085 vs 9,659)**			
Odds ratio (95% confidence intervals)	1.10 (0.96 - 1.26)	1.07 (0.95 - 1.21)	1.08 (0.95 - 1.21)
Meta-analysis P-value	0.17	0.24	0.23
I^2	0.531	0	0
Q	6.40	0.90	0.90
Sig. Q	1	1	1

Table S3 - Results for *CBLN2* and *PDE1A|DNAJC10* SNPs from the published GWAS in analyses from this study. I^2 **-** Heterogeneity index I2. Q; heterogeneity index, with calculated significance p-values (Sig. Q).

rsid	chr:pos	effect allele	other allele	effect allele frequency	OR	OR 95L	OR 95U	p	Bayes' factor	Posterior probabilities	rank
HLA-DPA1/DPB1 locus in all cohorts											
rs2071349	6:33043520	G	C	0.14	1.54	1.40	1.69	7.64E-20	4.49E+15	0.47	1
rs2856830	6:33041734	C	T	0.13	1.56	1.42	1.71	6.9E-20	3.80E+15	0.40	2
rs9277338	6:33032363	A	T	0.12	1.55	1.41	1.71	6.75E-19	4.66E+14	0.05	3
rs3830065	6:33037199	C	G	0.14	1.52	1.39	1.67	1.91E-18	2.56E+14	0.03	4
rs9277336	6:33030885	A	G	0.14	1.52	1.38	1.67	3.4E-18	1.56E+14	0.02	5
rs9277334	6:33030112	C	A	0.14	1.51	1.38	1.66	4.95E-18	1.15E+14	0.01	6
rs2301226	6:33034596	A	G	0.14	1.51	1.38	1.66	4.95E-18	1.14E+14	0.01	7
rs9277569	6:33058402	T	C	0.12	1.53	1.39	1.69	7.35E-18	6.14E+13	0.01	8
rs2295119	6:33060870	T	G	0.13	1.52	1.38	1.68	9.03E-18	5.58E+13	0.01	9
SOX17 signal 1 following conditioning on signal 2											
rs13266183	8:55267612	C	T	0.74	1.29	1.18	1.41	9.82E-09	1486027	0.46	1
rs12674755	8:55270204	C	T	0.74	1.28	1.17	1.40	2.13E-08	714438	0.22	2
rs10958403	8:55269940	G	A	0.74	1.28	1.17	1.39	2.87E-08	537646	0.17	3
rs12677277	8:55270271	T	C	0.73	1.27	1.17	1.39	3.22E-08	482807	0.15	4
SOX17 signal 2 following conditioning on signal 1											
rs9298503	8:55228897	C	T	0.92	1.65	1.42	1.92	4.16E-11	237193232	0.09	1
rs10103692	8:55258127	A	G	0.91	1.65	1.42	1.91	4.85E-11	205344048	0.08	2
rs12542396	8:55243761	G	T	0.91	1.64	1.41	1.90	6.23E-11	162151229	0.06	3
rs1868322	8:55253917	C	G	0.91	1.64	1.42	1.91	6.23E-11	161752712	0.06	4
rs10112815	8:55244463	A	G	0.91	1.64	1.41	1.90	6.46E-11	156760042	0.06	5
rs7816139	8:55242042	T	C	0.91	1.64	1.41	1.90	6.49E-11	155945033	0.06	6
rs12335302	8:55241412	G	A	0.91	1.64	1.41	1.90	6.58E-11	154044373	0.06	7.5
rs10958401	8:55241711	G	A	0.91	1.64	1.41	1.90	6.58E-11	154044373	0.06	7.5
rs7844284	8:55245449	C	T	0.91	1.64	1.41	1.90	7.14E-11	142573613	0.05	9
rs73598763	8:55262295	C	T	0.91	1.65	1.42	1.91	7.15E-11	141943947	0.05	10
rs10086131	8:55259753	T	A	0.92	1.64	1.41	1.90	9.5E-11	108440968	0.04	11
rs73598745	8:55250583	A	G	0.92	1.63	1.41	1.89	9.67E-11	106910852	0.04	12
rs4738801	8:55238494	G	C	0.91	1.63	1.40	1.88	1.18E-10	88684272	0.03	13
rs10106467	8:55257871	T	C	0.91	1.60	1.39	1.86	1.92E-10	56339958	0.02	14
rs10504164	8:55257452	A	G	0.91	1.60	1.39	1.85	2.08E-10	52155716	0.02	15
rs12676216	8:55257260	C	T	0.91	1.60	1.39	1.85	2.13E-10	51120007	0.02	16
rs12676167	8:55257035	C	A	0.91	1.60	1.39	1.85	2.2E-10	49587032	0.02	17
rs4738806	8:55263551	G	A	0.91	1.61	1.39	1.86	2.27E-10	48003796	0.02	18
rs12216711	8:55254302	C	T	0.91	1.60	1.38	1.85	2.49E-10	44173189	0.02	19
rs1992905	8:55253627	G	A	0.91	1.60	1.38	1.85	2.73E-10	40416575	0.01	20
rs61312536	8:55254404	C	T	0.91	1.60	1.38	1.85	2.75E-10	40117683	0.01	21.5
rs28656193	8:55254686	A	G	0.91	1.60	1.38	1.85	2.75E-10	40117683	0.01	21.5
rs12675939	8:55252939	A	G	0.91	1.60	1.38	1.85	2.78E-10	39820340	0.01	23
rs1354513	8:55252246	G	A	0.91	1.60	1.38	1.84	2.8E-10	39523830	0.01	24
rs765727	8:55251803	C	T	0.91	1.60	1.38	1.84	2.82E-10	39228639	0.01	25
rs1354512	8:55252228	C	T	0.91	1.60	1.38	1.84	2.83E-10	39064163	0.01	26.5
rs28584047	8:55255165	G	A	0.91	1.60	1.38	1.84	2.83E-10	39064163	0.01	26.5
rs2889135	8:55245138	G	C	0.91	1.59	1.38	1.84	3.16E-10	35225867	0.01	28
rs10504163	8:55255545	A	G	0.91	1.59	1.37	1.84	3.78E-10	29788342	0.01	29
rs77845050	8:55256491	G	A	0.91	1.59	1.37	1.84	3.81E-10	29568283	0.01	30
rs61290317	8:55264242	A	C	0.91	1.56	1.36	1.80	5.63E-10	20654788	0.01	31

Table S4 - Credible sets of variants with 99% of the posterior probability. Variants from each signal were analysed and assigned a likelihood of being the causal variant. OR, odds ratio, 95L, lower 95% confidence interval, 95U, upper 95% confidence interval, eaf, effect allele frequency, chr, chromosome, pos, position.

Group excluded		*HLA-DPA1/DPB1*: Lead SNP, rs2856830	*HLA-DPB1*: Missense SNP, rs1042140	*SOX17*: Lead SNP at signal 1, rs13266183	*SOX17*: Lead SNP at signal 2, rs10106467
**NIHRBR**					
BPD	OR (95% CI)	1.7 (1.47 - 1.96)	1.37 (1.21 - 1.54)	1.42 (1.24 - 1.62)	1.8 (1.43 - 2.26)
	Sig.	$3.56x10^{-13}$	$4.22x10^{-7}$	$2.37x10^{-7}$	$3.38x10^{-7}$
GEL	OR (95% CI)	1.68 (1.45 - 1.94)	1.36 (1.21 - 1.54)	1.47 (1.28 - 1.69)	1.83 (1.46 - 2.3)
	Sig.	$1.44x10^{-12}$	$4.76x10^{-7}$	$1.57x10^{-8}$	$1.18x10^{-7}$
Other	OR (95% CI)	1.75 (1.51 - 2.03)	1.36 (1.2 - 1.54)	1.45 (1.26 - 1.66)	1.81 (1.44 - 2.27)
	Sig.	$4.11x10^{-14}$	$6.43x10^{-7}$	$6.38x10^{-8}$	$2.64x10^{-7}$
PID	OR (95% CI)	1.66 (1.44 - 1.92)	1.39 (1.23 - 1.57)	1.44 (1.26 - 1.65)	1.83 (1.46 - 2.29)
	Sig.	$1.27x10^{-12}$	$6.15x10^{-8}$	$5.02x10^{-8}$	$1.04x10^{-7}$
SPEED	OR (95% CI)	1.68 (1.45 - 1.95)	1.36 (1.2 - 1.54)	1.44 (1.26 - 1.66)	1.83 (1.45 - 2.32)
	Sig.	$3.4x10^{-12}$	$9.79x10^{-7}$	$1.27x10^{-7}$	$2.84x10^{-7}$
BMPR2	OR (95% CI)	1.83 (1.57 - 2.12)	1.43 (1.26 - 1.63)	1.38 (1.2 - 1.59)	1.81 (1.42 - 2.3)
	Sig.	$4.6x10^{-16}$	$1.63x10^{-8}$	$5.07x10^{-6}$	$8.42x10^{-7}$
Pathogenic rare variants	OR (95% CI)	1.81 (1.56 - 2.10)	1.42 (1.25 - 1.62)	1.41 (1.22 - 1.63)	1.81 (1.42 - 2.31)
	Sig.	$5.8x10^{-15}$	$8.24x10^{-8}$	$2.43x10^{-6}$	$1.95x10^{-6}$
**PAHB**					
T1D	OR (95% CI)	1.47 (1.21-1.78)	1.42 (1.21-1.66)	1.56 (1.32-1.84)	1.78 (1.35-2.36)
	Sig.	$1.10x10^{-4}$	$1.88x10^{-5}$	$2.07x10^{-7}$	$5.09x10^{-5}$
CTD	OR (95% CI)	1.47 (1.21-1.77)	1.41 (1.20-1.64)	1.51 (1.28-1.78)	1.79(1.36-2.35)
	Sig.	$8.03x10^{-5}$	$2.11x10^{-5}$	$7.62x10^{-7}$	$3.68x10^{-5}$
T1D and CTD	OR (95% CI)	1.50 (1.23-1.83)	1.43 (1.22-1.68)	1.55 (1.31-1.83)	1.79 (1.35-2.38)
	Sig.	$5.51x10^{-5}$	$1.46x10^{-5}$	$4.56x10^{-7}$	$5.10x10^{-5}$
BMPR2	OR (95% CI)	1.49 (1.22-1.80)	1.40 (1.19-1.65)	1.57 (1.32-1.87)	1.94 (1.44-2.61)
	Sig.	$6.27x10^{-5}$	$4.487x10^{-5}$	$1.84x10^{-7}$	$1.10x10^{-5}$
Pathogenic rare variants	OR (95% CI)	1.50 (1.24- 1.83)	1.42 (1.21-1.67)	1.59 (1.34-1.88)	1.94 (1.44-2.62)
	Sig.	$4.07x10^{-5}$	$2.22x10^{-5}$	$1.44x10^{-7}$	$1.30x10^{-5}$

Table S5 - Sensitivity analyses in UK NIHRBR study and US PAHB study **-** association results are shown for main SNPs of interest after exclusion of each of the main control disease groups, or PAH cases with pathogenic *BMPR2* rare variants.

Variable name	T/T	T/C	C/C	Sig.	q
NIHRBR					
n [%]	559 [66.0%]	242 [28.6%]	46 [5.4%]		
Sex: female (n [%])	359 [64.2%]	182 [75.2%]	38 [82.6%]	<0.001	<0.001
Age at diagnosis (years)	52.7 [39.2 - 66.3]	48.2 [35.8 - 61.0]	43.5 [32.4 - 59.9]	0.001	0.048
Vasoresponder (n [%])	16 [12.3%]	7 [13.5%]	3 [16.7%]	0.615	0.869
mPAP (mmHg)	53.0 [44.0 - 61.0]	53.0 [44.0 - 62.0]	53.0 [43.0 - 61.0]	1	1
PCWP (mmHg)	10.0 [7.0 - 12.0]	9.0 [7.0 - 12.0]	10.0 [7.0 - 14.0]	0.835	0.968
CO (L/min)	3.9 [3.2 - 5.0]	4.0 [3.2 - 4.9]	4.6 [3.8 - 4.8]	0.394	0.805
CI (L/min/m2)	2.1 [1.7 - 2.6]	2.2 [1.7 - 2.8]	2.4 [2.0 - 2.6]	0.072	0.405
PVR (WU)	11.1 [7.3 - 15.1]	11.2 [7.5 - 14.7]	9.2 [7.8 - 13.5]	0.627	0.869
Functional class, (n [%]) : I	12 [2.4%]	5 [2.5%]	0 [0.0%]	0.121	0.548
II	96 [19.3%]	52 [26.0%]	15 [36.6%]		
III	335 [67.4%]	121 [60.5%]	21 [51.2%]		
IV	54 [10.9%]	22 [11.0%]	5 [12.2%]		
6mwt distance (m)	300.0 [148.5 - 396.4]	356.0 [248.0 - 426.0]	316.5 [228.8 - 366.2]	0.046	0.361
PAHB					
Sex, Female	373 [77.7%]	147 [79.0%]	19 [67.9%]	0.628	
Age, years	53.5 [41.1 - 63.4]	49.2 [37.2 - 59.1]	44.3 [32.0 - 58.1]	0.004	
mPAP (mmHg)	51.0 [41.0 - 59.0]	52.0 [41.3 - 60.0]	54.0 [43.0 - 59.5]	0.665	
PCWP (mmHg)	10.0 [7.0 - 13.0]	10.0 [7.8 - 12.3]	11.0 [8.0 - 12.0]	0.233	
CO (L/min)	4.3 [3.4 - 5.2]	4.5 [3.6 - 5.2]	4.2 [3.6 - 4.9]	0.925	
CI (L/min/m2)	2.2 [1.8 - 2.8]	2.4 [1.8 - 2.8]	2.2 [1.9 - 2.4]	0.515	
PVR (WU)	9.8 [6.4 - 14.4]	9.7 [6.4 - 13.7]	9.7 [7.3 - 11.7]	0.633	
Functional class, (n [%]) I	15 [5.3%]	5 [4.4%]	2 [12.5%]	0.142	
II	71 [25.1%]	43 [37.7%]	4 [25.0%]		
III	177 [62.5%]	57 [50.0%]	9 [56.2%]		
IV	20 [7.1]	9 [7.9%]	1 [6.3%]		
6mwt distance (m)	344.2 [270.8 - 434.2]	348.5 [230.8 - 425.6]	337.1 [269.8 - 399.1]	0.046	
PHAAR					
Sex, Female	149 [72.0%]	62 [63.9%]	12 [80.0%]	0.557	
Age, years	50.4 [34.5 - 60.0]	49.2 [36.8 - 58.5]	41.7 [32.4 - 49.2]	0.334	
BHFPAH					
Sex, Female	127 [68.3%]	54 [63.5%]	3 [75%]	0.700	
Age, years	53.6 [37.9 - 68.5]	48.6 [35.4 - 61.7]	32.6 [30.8 - 49.5]	0.137	

Table S6 - Characteristics of PAH patients with different HLA lead SNP rs2856830 genotype

UK	Europeans in UK NIHRBR		
rs2856830 genotype	T/T	T/C	C/C
Diabetes Mellitus	35	14	4
Coronary Heart Disease	40	8	1
Systemic Hypertension	23	14	3
Hyperlipidaemia	21	7	1
Autoimmune disorders	19	3	1
Negative autoantibodies tests	237	91	23
Autoantibodies detected	64	26	2
Total	577	243	44
As percentages			
Diabetes Mellitus	6%	6%	9%
Coronary Heart Disease	7%	3%	2%
Systemic Hypertension	4%	6%	7%
Hyperlipidaemia	4%	3%	2%
Autoimmune disorders	3%	1%	2%
Negative autoantibodies tests	41%	37%	52%
Autoantibodies detected	11%	11%	5%
AutoAb of those tested	21%	22%	8%

Table S7 - Count and percentages of comorbidities in UK study individuals divided by HLA lead variant rs2856830 genotype

Europeans			Frequency by rs2856830 genotype					Frequency in PAH vs non-PAH controls			
Family	Gene	Type	T/T	T/C	C/C	Sig.	q FDR	Controls	Cases	Sig	q FDR
HLA	DPB1	02	0.03	0.48	0.97	<5e-247	<5e-247	0.138	0.208	4.61E-14	8.64E-11
HLA	DPB1	0201	0.03	0.44	0.90	<5e-247	<5e-247	0.129	0.197	7.63E-13	7.15E-10
HLA	DPB1	04	0.61	0.32	0.00	1.69E-243	6.34E-241	0.539	0.485	0.00054	0.0365
HLA	DPB1	0401	0.48	0.26	0.00	1.28E-140	2.40E-138	0.425	0.390	0.0285	0.217
HLA	DPB1	0202	0.00	0.03	0.07	1.63E-89	2.77E-87	0.009	0.011	0.686	0.902
HLA	DPA1	0103	0.79	0.90	1.00	1.07E-50	6.23E-49	0.815	0.831	0.505	0.843
HLA	DPA1	02	0.21	0.09	0.00	1.57E-50	8.40E-49	0.179	0.165	0.555	0.869
HLA	DPA1	01	0.79	0.91	1.00	1.80E-49	8.44E-48	0.821	0.835	0.579	0.882
HLA	DPA1	0201	0.17	0.07	0.00	7.14E-46	3.26E-44	0.146	0.130	0.406	0.774
HLA	DPB1	16	0.00	0.02	0.02	1.70E-42	7.08E-41	0.006	0.008	0.155	0.571
HLA	DPB1	1601	0.00	0.02	0.02	1.70E-42	7.08E-41	0.006	0.008	0.155	0.571
HLA	DPB1	0402	0.13	0.06	0.00	5.12E-25	2.08E-23	0.115	0.095	0.109	0.478
HLA	DPB1	03	0.12	0.06	0.00	1.44E-24	5.50E-23	0.105	0.111	0.205	0.647
HLA	DPB1	0301	0.12	0.06	0.00	1.44E-24	5.50E-23	0.105	0.111	0.205	0.647
HLA	DRB1	0302	0.00	0.00	0.01	1.80E-12	6.14E-11	0.000	0.000	0.657	0.898
HLA	DRB1	0406	0.00	0.00	0.01	6.65E-12	2.19E-10	0.001	0.000	0.604	0.884
HLA	DPB1	01	0.07	0.04	0.00	3.76E-10	1.17E-08	0.061	0.055	0.744	0.915
HLA	DPB1	0101	0.07	0.04	0.00	3.76E-10	1.17E-08	0.061	0.055	0.744	0.915
HLA	DRB1	10	0.00	0.01	0.01	5.31E-08	1.44E-06	0.006	0.006	0.769	0.922
HLA	DRB1	1001	0.00	0.01	0.01	5.31E-08	1.44E-06	0.006	0.006	0.769	0.922
HLA	DRB1	0103	0.00	0.00	0.02	5.74E-08	1.53E-06	0.002	0.001	0.497	0.840
HLA	DRB1	03	0.05	0.08	0.10	6.53E-07	1.37E-05	0.060	0.062	0.863	0.958
HLA	DRB1	0301	0.05	0.08	0.10	9.58E-07	1.93E-05	0.059	0.061	0.884	0.966
HLA	B	18	0.03	0.05	0.09	1.21E-06	2.38E-05	0.037	0.055	0.00105	0.0463
HLA	B	1801	0.03	0.05	0.09	1.21E-06	2.38E-05	0.037	0.055	0.00105	0.0463
HLA	DRB1	1502	0.00	0.01	0.02	3.74E-06	7.08E-05	0.004	0.004	0.841	0.950

Table S8 - Association of HLA types with lead GWAS HLA variant rs2856830 and PAH. Frequencies of HLA types are shown for all subjects by rs2856830 genotype and by non-PAH controls and PAH cases with p-values from chi-squared tests, raw and FDR-corrected.

				In Europeans selected for UK GWAS (n=5895) Frequencies and significance by chi-squared test			
Family	Gene	Allele	digits	non-PAH	PAH	Sig.	p. FDR
HLA	DPB1	02	2	0.14	0.21	1.17E-14	4.95E-12
HLA	DPB1	02:01	4	0.13	0.20	2.31E-13	4.91E-11
HLA	B	35:01	4	0.05	0.06	2.49E-05	0.00352
HLA	DRB1	15:01	4	0.07	0.09	0.0003	0.0180
HLA	DPB1	04	2	0.54	0.48	0.0002	0.0130
HLA	B	35	2	0.07	0.09	0.0002	0.0130
HLA	B	50:01	4	0.01	0.01	0.0014	0.0486
HLA	DRB1	15	2	0.08	0.10	0.0004	0.0184
HLA	B	50	2	0.01	0.01	0.0014	0.0486
HLA	B	18	2	0.04	0.06	0.0004	0.0181
HLA	B	18:01	4	0.04	0.06	0.0004	0.0181
HLA	B	44	2	0.17	0.13	0.0002	0.0130
HLA	C	15	2	0.02	0.03	0.0015	0.0492

Table S9 - HLA types associated with PAH in Europeans after FDR-correction.

Associations with *HLA-DPB1* alleles

Position / Allele	8	9	11	33	35	36	55	56	57	65	69	76	84	85	86	87	96	178	194
*DPB1**02:01	L	F	G	E	F	V	D	E	E	I	E	M	G	G	P	M	R	L	R
*DPB1**02:02	L	F	G	E	L	V	E	A	E	I	E	M	G	G	P	M			
*DPB1**16:01	L	F	G	E	F	V	D	E	E	I	E	M	D	E	A	V			
*DPB1**03:01	V	Y	L	E	F	V	D	E	D	L	K	V	D	E	A	V	K	L	R
*DPB1**04:01	L	F	G	E	F	A	A	A	E	I	K	M	G	G	P	M	R	L	R
*DPB1**04:02	L	F	G	E	F	V	D	E	E	I	K	M	G	G	P	M	R	M	R
*DPB1**01:01	V	Y	G	E	Y	A	A	A	E	I	K	V	D	E	A	V	K	L	Q

Position / Allele	Frequencies by GWAS SNP rs2856830			Association with PAH			Survival analysis		
	T/T	T/C	C/C	non-PAH	PAH	Sig	Hazard ratio	95% CI	Sig
*DPB1**02:01	3%	44%	90%	13%	20%	$8x10^{-13}$	0.70	0.49 -	0.049
*DPB1**02:02	0%	3%	7%	0.9%	1.1%	0.69	0.31	0.04 -	0.243
*DPB1**16:01	0%	2%	2%	0.6%	0.8%	0.16	1.29	0.41 -	0.667
*DPB1**03:01	12%	6%	0%	10%	11%	0.20	0.88	0.59 -	0.550
*DPB1**04:01	48%	26%	0%	43%	39%	0.029	1.33	1.04 -	0.026
*DPB1**04:02	13%	6%	0%	11%	10%	0.11	1.17	0.81 -	0.401
*DPB1**01:01	7%	4%	0%	6.1%	5.5%	0.74	0.81	0.46 -	0.461

Associations with specific *HLA-DPB1* residues

Allele / Position	Amino acid residues in *DPB1* alleles							Frequencies by GWAS SNP rs2856830								Survival analysis							
	02:01	02:02	16:01	03:01	04:01	04:02	01:01	Residue	T/T	T/C	C/C	Residue	T/T	T/C	C/C	Residue	Hazard ratio	95% CI	Sig	Residue	Hazard ratio	95% CI	Sig
36	V	V	V	V	A	V	A	A	60%	32%	1%					A	1.34	1.05 - 1.72	0.021				
55	D	E	D	D	A	D	A	A	60%	33%	0%	D	37%	62%	92%	A	1.33	1.04 - 1.71	0.024	D	0.77	0.6 - 0.99	0.044
56	E	A	E	E	A	E	A	A	63%	36%	9%					A	1.29	1.01 - 1.66	0.044				
69	E	E	E	K	K	K	K	K	84%	44%	1%	E	12%	55%	99%	K	1.37	1.05 - 1.8	0.022	E	0.71	0.54 - 0.94	0.018

Table S10 - Associations of *HLA-DPB1* alleles and specific amino acid residues with the lead *HLA-DPB1* SNP rs2856830, diagnosis of PAH and survival in PAH. Orange indicates alleles and residues depleted in PAH cases and green indicates those enriched in PAH cases. Green to red shading of percentages and hazard ratios is used to indicate directionality of associations (green indicates enriched in genotype/associated with improved outcomes in PAH).

Guide RNA	Sequence	Hg19 target locus	Species
Negative control 1: Blue fluorescent protein (BFP)#	BFP CDS guide 1:<br>ATGGCGTGCAGTGCTTCAGC	NA	Human
Negative control 2: Green fluorescent protein (eGFP)#	eGFP CDS guide 1:<br>GAAGTTCGAGGGCGACACCC	NA	Human
Negative control 3: Area upstream of *SOX17*	F: CACCGTATGTTCCCTAGCCAAGACT<br>R: AAACAGTCTTGGCTAGGGAACATAC	chr8:55353563-55353583	Human
Positive control: *SOX17* promoter	F: CACCGTCTGGTCTACAGCGTACCC<br>R: AAACGGGTACGCTGTAGACCAGAC	chr8: 55370524-55370543	Human
GWAS locus guide 1	F: CACCGAAGGCCTCCCCAATTGTGTA<br>R: AAACTACACAATTGGGGAGGCCTTC	chr8:55269985-55270007	Human
GWAS locus guide 2	F: CACCGTAAGCCATACACAATTGGGG<br>R: AAACCCCCAATTGTGTATGGCTTAC	chr8:55269992-55270012	Human
GWAS locus guide 3	F: CACCGTACTGGAGGCCCACAATGTG<br>R: AAACCACATTGTGGGCCTCCAGTAC	chr8:55270017-55270037	Human

qPCR Primers (gene)	Sequence	Species
SOX17	F: GGACCGCACGGAATTTGAAC<br>R: GGACACCACCGAGGAAATGG	Human
MRPL15	F: GCGGATCCTGCCAAATTTCC<br>R: AACTCTGCTTCCTTGGACGG	Human
TMEM68	F: CAGCCGTTTGGCATGGTTAT<br>R: GCGATGACCCCATACGATGT	Human
ACTB1	F: GCACCACACCTTCTACAATGA<br>R: GTCATCTTCTCGCGGTTGGC	Human

Primers for cloning of 100bp region	Sequence	Species
rs1095403	F: ctggcctaactggccggtacAAATAGAAGCGACGCTGC<br>R: aggctagcgagctcaggtacCAATTGGGGAGGCCTTTTG	Human
rs12674755	F: ctggcctaactggccggtacGGACAGCCACCCATTTTATC<br>R: aggctagcgagctcaggtacACCTCCCTTTAAGCTTAATTC	Human
rs12677277	F: ctggcctaactggccggtacACAGGCTGGAGGCACAGTC<br>R: aggctagcgagctcaggtacCAAGTGCCAGGCACTTCC	Human
rs765727	F: ctggcctaactggccggtacTGGGTCCTGGTCTGGATG<br>R: aggctagcgagctcaggtacGGATGAGTCTGATGGCTC	Human

Table S11 – Oligonucleotide sequences used as guide RNA in CRISPR inhibition experiments and as primers for quantitative PCR. Testing for primer efficiency demonstrated primer efficiencies between 90 and 110%. Uppercase: Gene-specific primer. BFP = Blue fluorescent protein, F = Forward, GFP = Green fluorescent protein, NA = not applicable, R = Reverse. #negative control guides were taken from the Addgene validated guide RNA repository https://www.addgene.org/crispr/validated-grnas/ accessed 01/07/2017.

Supplementary Figures

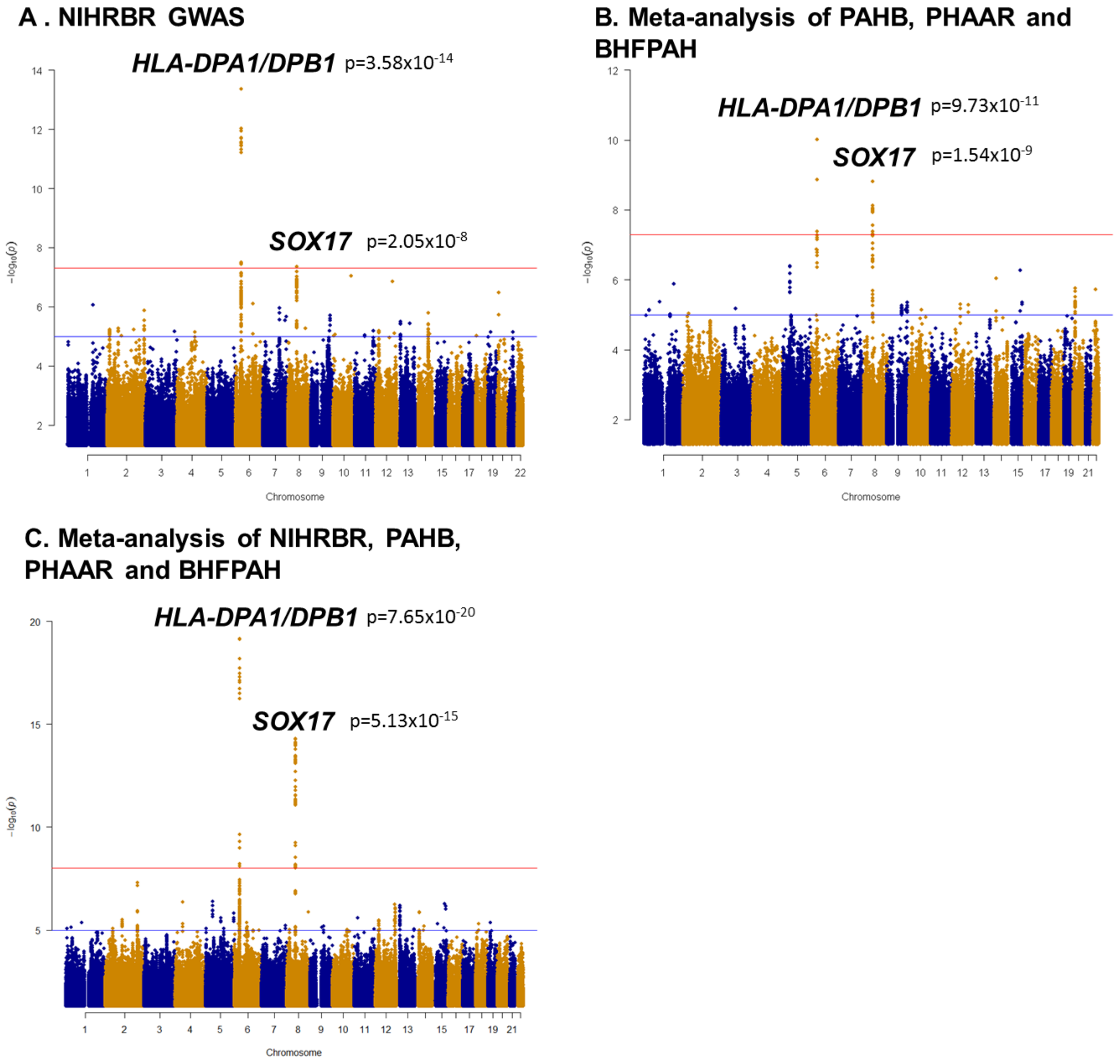

Figure S1 - Genome-wide association analysis results, illustrated by Manhattan plots. **A.** GWAS of WGS data from NIHRBR and **B**. Meta-analysis of genome-wide genotype data from PAHB, PHAAR and BHFPAH. **C**. Meta-analysis of all four studies.

A. Fine mapping and functional characterisation of *SOX17* locus

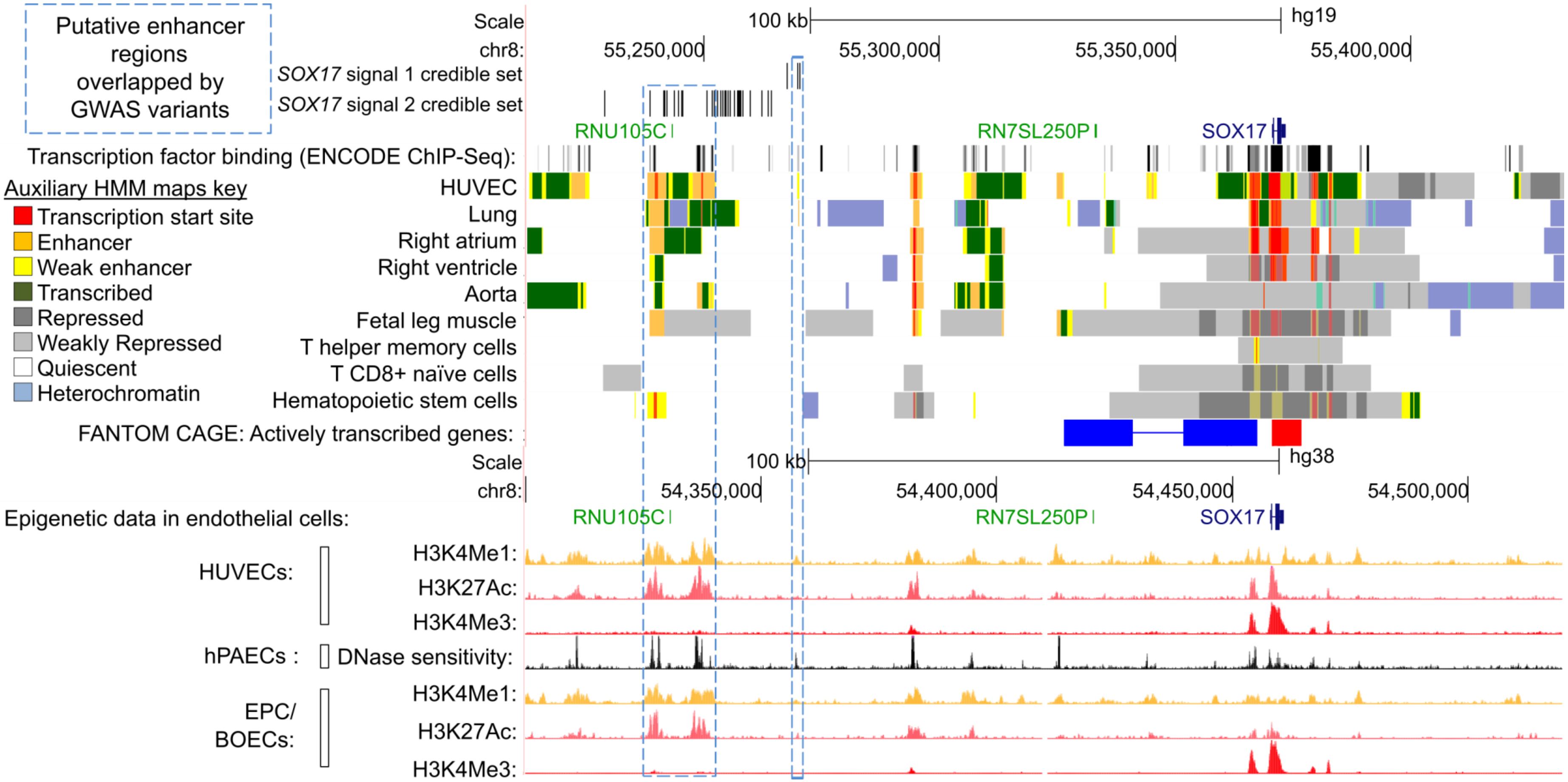

B. Genomic regions spatially associated with *SOX17* promoter in circulating endothelial progenitor cells: promoter-capture Hi-C

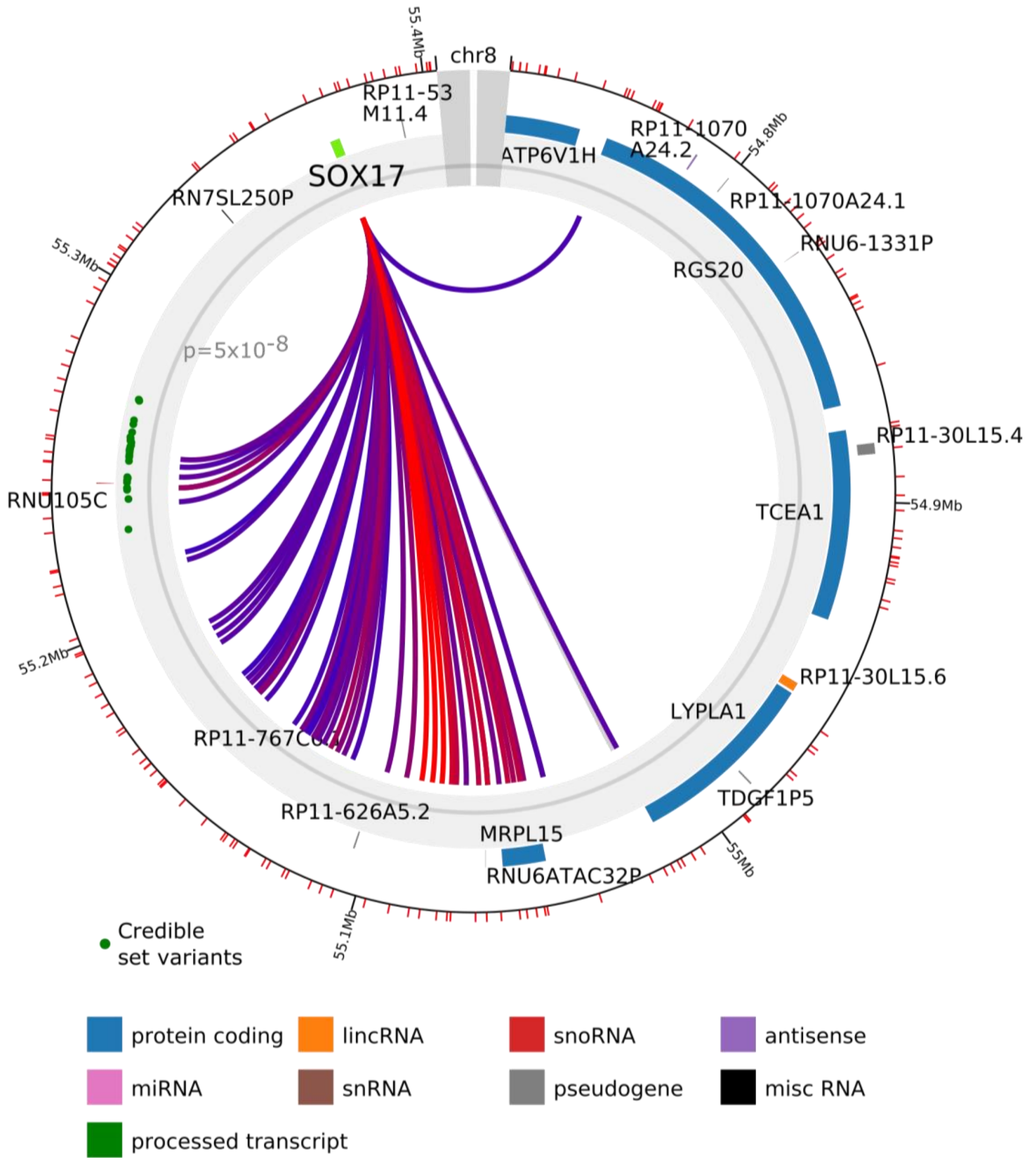

Figure S2 - Annotation of the *SOX17* locus. **A.** Mapping of *SOX17* locus variants associated with PAH with public epigenetic data. The credible sets for signals 1 and 2 indicate positions of variants 99% likely to contain the causal variants. *RNU105C* and *RN7SL250P* are non-coding RNA genes and the location of the *SOX17* gene is shown. Transcription factor binding sites as determined by ChIP-Seq experiments of 161 factors from ENCODE with Factorbook Motifs are shown. Auxiliary hidden markov models (HMM), which summarise epigenetic data to predict the functional status of genomic regions in different tissues/cells are shown. FANTOM Cap Analysis of Gene Expression (CAGE) data indicate actively transcribed genes with stringent criteria. Epigenetic data in endothelial cells (EC) including human umbilical vein ECs (HUVEC), human pulmonary artery ECs (hPAECs) and endothelial progenitor cells (EPC), also known as blood outgrowth ECs (BOEC) included indicate areas likely to contain active regulatory regions and promoters. Markers include histone 3 lysine 4 monomethylation (H3K4Me1, often found in enhancers) and trimethylation (H3K4Me3 strongly observed in promoters) and lysine 27 acetylation (often found in active regulatory regions). The blue dashed line indicates the area where epigenetic data suggest a putative enhancer region, overlapped by

variants associated with PAH. **B.** Promoter capture Hi-C data for the *SOX17* promoter from chicp.org plotter in endothelial precursor cells. Lines indicate significant associations between genomic loci. Green dots indicate positions of variants in *SOX17* locus credible set (99% likely to contain causal variant), their distance from the centre of the figure indicates the strength of their association with PAH; the dark grey line indicates genomewide significance.

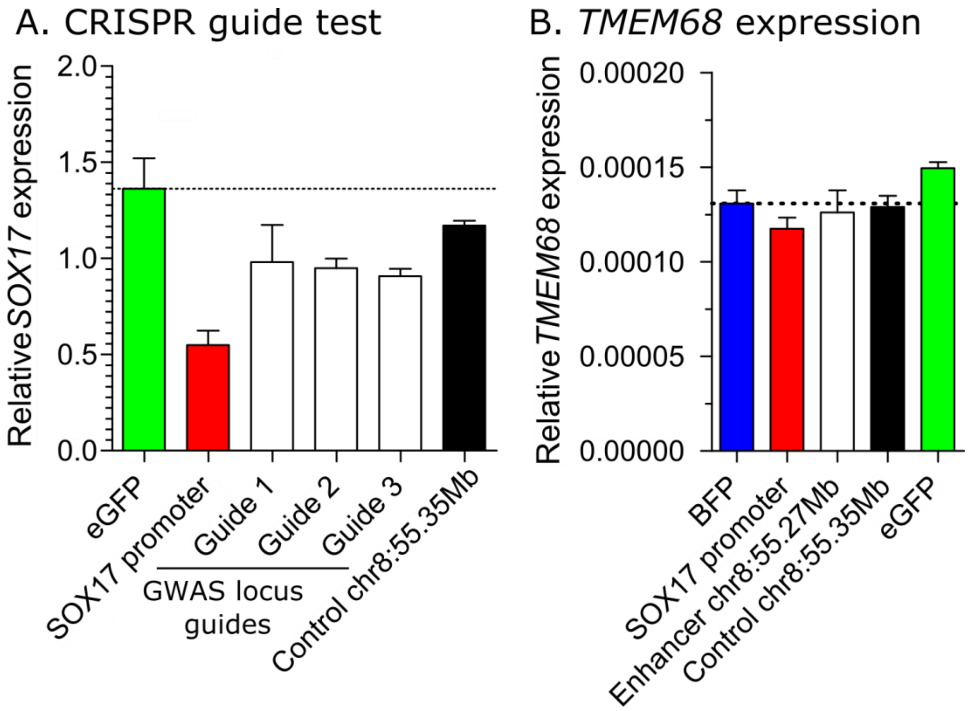

Figure S3 - A. Screening for the repression efficiency of three different guides against the region around the GWAS lead SNP by relative Sox17 mRNA expression, showing the most efficient repression with guide RNA 3, as compared to negative controls. Mean±SEM of n=2-4 experiments. **B.** Expression of a gene 1.3Mb 3' to *SOX17, TMEM68* in representative CRISPR repression experiment with n=4 replicates.

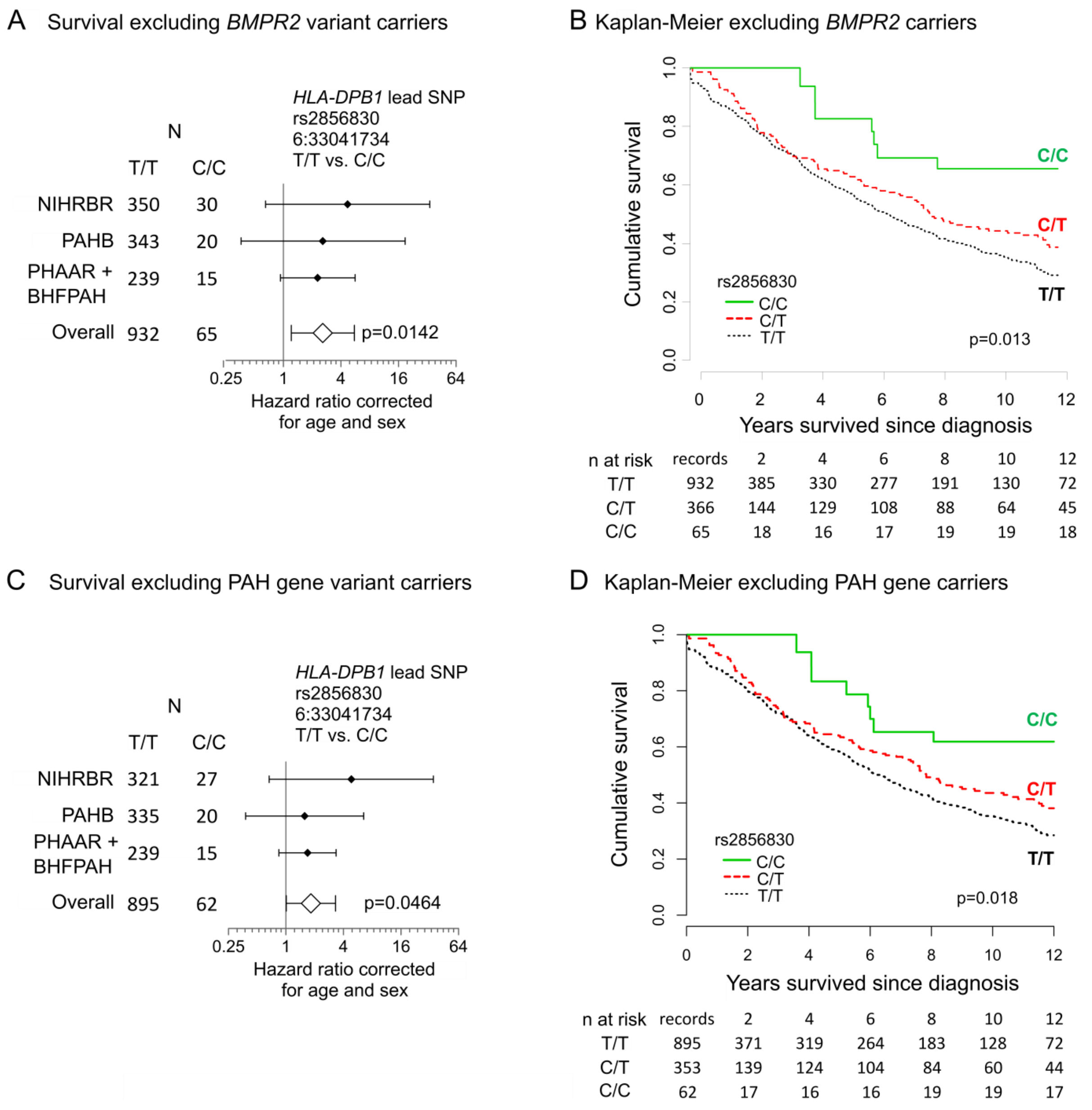

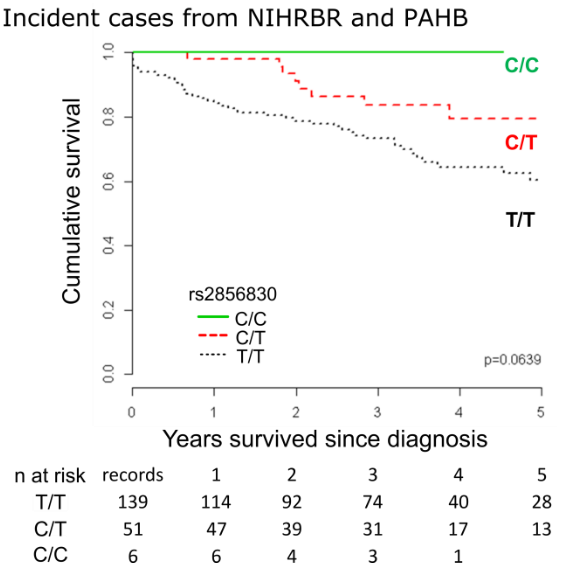

Figure S4 - Survival sensitivity analyses. **A.** Meta-analysis of all cohorts for homozygosity at the *HLA-DPB1* lead SNP excluding *BMPR2* mutation carriers. **B.** Kaplan-Meier analysis excluding *BMPR2* mutation carriers. **C.** Meta-analysis of all cohorts for homozygosity at the *HLA-DPB1* lead SNP excluding *rare, pathogenic* mutation carriers. **D.** Kaplan-Meier analysis excluding *rare, pathogenic* mutation carriers. **E.** Kaplan-Meier survival estimate curves for sub-analysis of incident NIHRBR/PAHB PAH cases (sampled for DNA testing within 6 months of diagnosis).

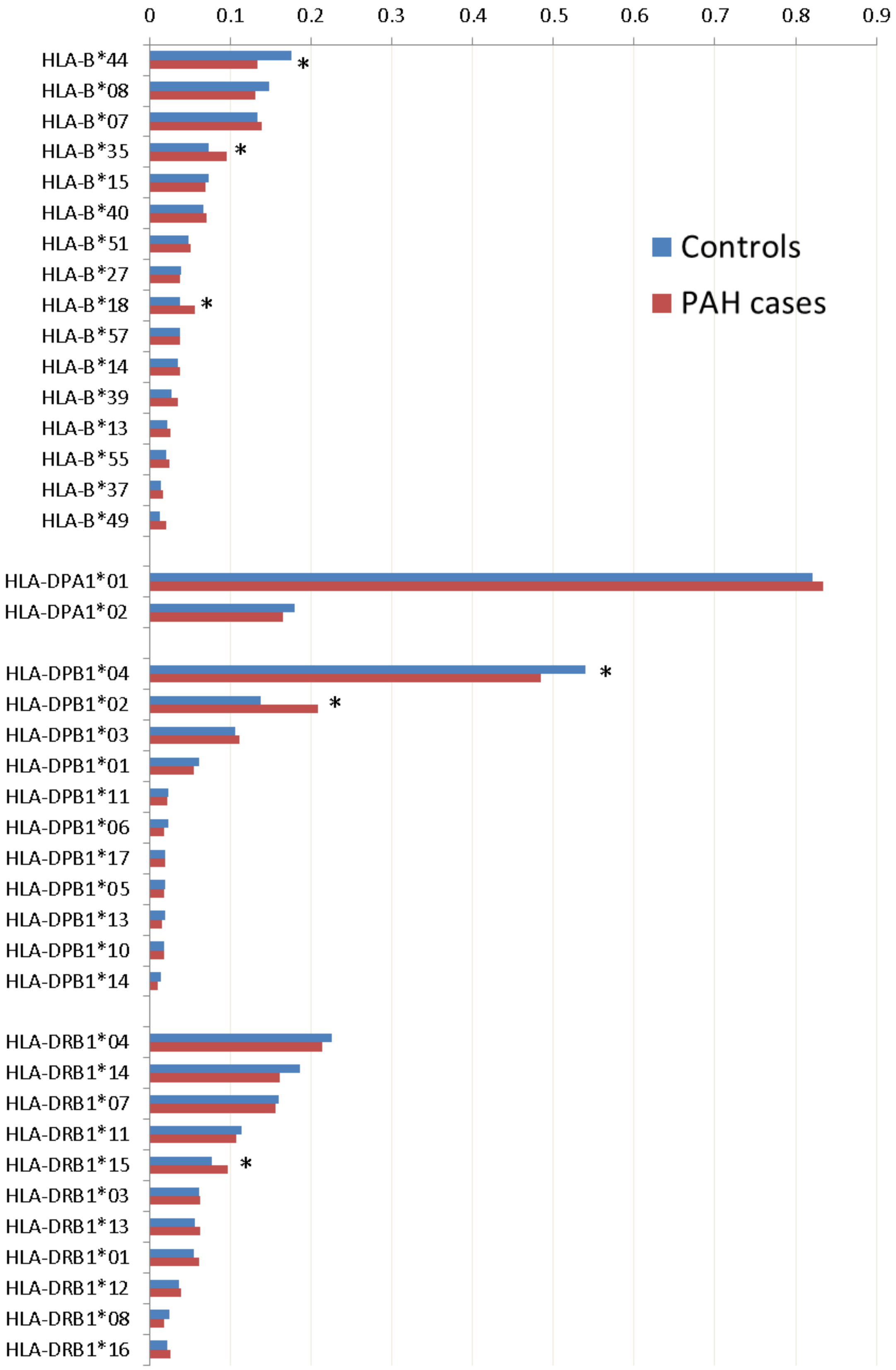

Figure S5 - Frequencies of HLA allele groups in NIHRBR controls and PAH cases. * indicates significance after FDR correction $p<0.05$. Only HLA B, DPA1, DPB1 and DRB1 allele groups with more than 1% frequency in controls are shown.

Table legends

Table S1 - Genotyping details and quality controls of studies

Table S2 - Heterogeneity and imputation quality of lead SNPs across studies. I^2 - Heterogeneity index I2.

Table S3 - Results for *CBLN2* SNP from the published Paris GWAS, rs2217560, in GWAS analyses from this study.

Table S4 - Credible sets of variants with 99% of the posterior probability. Variants from each signal were analysed and assigned a likelihood of being the causal variant.

Table S5 - Sensitivity analyses in UK NIHRBR study - association results are shown for main SNPs of interest after exclusion of each of the main control disease groups, or PAH cases with pathogenic *BMPR2* rare variants.

Table S6 - Characteristics of PAH patients with different HLA lead SNP rs2856830 genotype

Table S7 - Count and percentages of comorbidities in BRIDGE subjects divided by HLA lead variant

Table S8 - Association of HLA types with lead GWAS HLA variant and PAH. Frequencies of HLA types are shown for all subjects by genotype of lead GWAS variant, and divided by non-PAH controls and PAH cases with p-values from chi-squared tests, raw and FDR-corrected.

Table S9 - HLA types associated with PAH in Europeans after FDR-correction.

Table S10 - Associations of HLA-DPB1 alleles and specific amino acid residues with the lead HLA-DPB1 SNP rs2856830, diagnosis of PAH and survival in PAH. Orange indicates alleles and residues depleted in PAH cases and green indicates those enriched in PAH cases. Green to red shading of percentages and hazard ratios is used to indicate directionality of associations (green indicates enriched in genotype/associated with improved outcomes in PAH).

Table S11 – Oligonucleotide sequences used as guide RNA in CRISPR inhibition experiments and as primers for quantitative PCR. Testing for primer efficiency demonstrated primer efficiencies between 90 and 110%. BFP = Blue fluorescent protein, F = Forward, GFP = Green fluorescent protein, NA = not applicable, R = Reverse. #negative control guides were taken from the Addgene validated guide RNA repository https://www.addgene.org/crispr/validated-grnas/ accessed 01/07/2017.

Figure legends

Figure S1 - Genome-wide association analysis results, illustrated by Manhattan plots. A. UK WGS study NIHRBR and B. Genotyping studies including PAHB, PHAAR and BHFPAH. C. Meta-analysis of all four studies.

Figure S2 - Annotation of the *SOX17* locus. A. Mapping of *SOX17* locus variants associated with PAH with public epigenetic data. GWS variants indicates positions of specific variants which reached p<5x10-8 in the discovery analysis in the UK and US studies. The credible sets indicate positions of variants 99% likely to contain the causal variants. *RNU105C* and *RN7SL250P* are non-coding RNA genes and the location of the *SOX17* gene is shown. Transcription factor binding sites as determined by ChIP-Seq experiments of 161 factors from ENCODE with Factorbook Motifs are shown. Auxiliary hidden markov models (HMM), which summarise epigenetic data to predict the functional status of genomic regions in different tissues/cells are shown. FANTOM Cap Analysis of Gene Expression (CAGE) data indicate actively transcribed genes with stringent criteria. Epigenetic data in endothelial cells (EC) including human umbilical vein ECs (HUVEC), human pulmonary artery ECs (hPAECs) and endothelial progenitor cells (EPC), also known as blood outgrowth ECs (BOEC) included indicate areas likely to contain active regulatory regions and promoters. Markers include histone 3 lysine 4 monomethylation (H3K4Me1, often found in enhancers) and trimethylation (H3K4Me3 strongly observed in promoters) and lysine 27 acetylation (often found in active regulatory regions). The blue dashed line indicates the area where epigenetic data suggest a putative enhancer region, overlapped by variants associated with PAH. B. Promoter capture Hi-C data for the *SOX17* promoter from chicp.org plotter in endothelial precursor cells. Lines indicate significant associations between genomic loci. Green dots indicate positions of variants in *SOX17* locus credible set (99% likely to contain causal variant), their distance from the centre of the figure indicates the strength of their association with PAH; the dark grey line indicates genomewide significance.

Figure S3 - A. Screening for the repression efficiency of three different guides against the region around the GWAS lead SNP by relative Sox17 mRNA expression, showing the most efficient repression with guideRNA 3, as compared to negative controls. Mean±SEM of n=2-4 experiments. B. Expression of a gene 1.3Mb 3’ to SOX17, TMEM68 in representative CRISPR repression experiment with n=4 replicates.

Figure S4 - Survival sensitivity analyses. A. Meta-analysis of all cohorts for homozygosity at the *HLA-DPB1* lead SNP excluding *BMPR2* mutation carriers. B. Kaplan-Meier analysis excluding *BMPR2* mutation carriers. C. Meta-analysis of all cohorts for homozygosity at the *HLA-DPB1* lead SNP excluding *rare, pathogenic* mutation carriers. D. Kaplan-Meier analysis excluding *rare, pathogenic* mutation carriers. E. Kaplan-Meier survival estimate curves for sub-analysis of incident NIHRBR/PAHB PAH cases (sampled for DNA testing within 6 months of diagnosis).

Figure S5 - Frequencies of HLA allele groups in UK controls and PAH cases. * indicates significance after FDR correction p<0.05. Only HLA B, DPA1, DPB1 and DRB1 allele groups with more than 1% frequency in controls are shown.

Full authorship and affiliations details:

Authors:

Christopher J. Rhodes, Ph.D.* [1], Ken Batai, Ph.D.* [2], Marta Bleda, Ph.D.* [3], Matthias Haimel, B.Sc.* [3,4,5], Laura Southgate, Ph.D.* [6,7], Marine Germain, Ph.D.* [8], Michael W. Pauciulo, B.S., M.B.A.* [9], Charaka Hadinnapola, M.B. B.Chir. [3], Jurjan Aman, M.D., Ph.D. [1], Barbara Girerd, Ph.D. [10], Amit Arora, MD MPH [2], Jo Knight, Ph.D. [11], Ken B. Hanscombe, Ph.D. [7], Jason H. Karnes, PharmD, PhD [12], Marika Kaakinen, Ph.D. [1], Henning Gall, M.D., Ph.D. [13], Anna Ulrich, MSc [1], Lars Harbaum, M.D. [1], Inês Cebola, Ph.D. [14], Prof. Jorge Ferrer, Ph.D. [14], Katie Lutz, B.S. [9], Emilia M. Swietlik, M.D. [3], Prof. Ferhaan Ahmad, M.D., Ph.D. [15], Prof. Philippe Amouyel, M.D, Ph.D. [16], Prof. Stephen L. Archer, M.D. [17], Rahul Argula, M.D. [18], Eric D. Austin, M.D. [19], Prof. David Badesch, M.D. [20], Sahil Bakshi, DO [21], Christopher F. Barnett, M.D. [22], Prof. Raymond Benza, M.D. [23], Nitin Bhatt, M.D. [24], Harm J. Bogaard, M.D., Ph.D. [25], Prof. Charles D. Burger, M.D. [26], Murali M. Chakinala, M.D. [27], Colin Church, Ph.D. [28], John G. Coghlan, M.D. [29], Robin Condliffe, M.D. [30], Prof. Paul A. Corris, M.B.B.S. [31], Prof. Cesare Danesino, M.D. [32,33], Prof. Stéphanie Debette, M.D, Ph.D. [34], Prof. C. Gregory Elliott, M.D. [35], Prof. Jean Elwing, M.D. [36], Melanie Eyries, Ph.D. [8], Terry Fortin, M.D. [37], Prof. Andre Franke, Ph.D. [38], Prof. Robert P. Frantz, M.D. [39], Prof. Adaani Frost, M.D. [40], Prof. Joe G. N. Garcia, M.D. [41], Prof. Stefano Ghio, M.D. [33], Prof. Hossein-Ardeschir Ghofrani, M.D. [13,1], J. Simon R. Gibbs, M.D. [42], Prof. John B. Harley, M.D., Ph.D. [43,79], Hua He, Ph.D. [9], Prof. Nicholas S. Hill, M.D. [44], Prof. Russel Hirsch, M.D. [45], Arjan C. Houweling, M.D., Ph.D. [25], Luke S. Howard, M.D., Ph.D. [42], Prof. Dunbar Ivy, M.D. [46], Prof. David G. Kiely, M.D. [30], Prof. James Klinger, M.D. [47], Gabor Kovacs, M.D. [48,49], Tim Lahm, M.D. [50], Prof. Matthias Laudes, M.D. [51], Rajiv D. Machado, Ph.D. [52], Robert V. MacKenzie Ross, M.B. B.Chir. [53], Keith Marsolo, Ph.D. [54], Prof. Lisa J. Martin, Ph.D. [9], Shahin Moledina, M.B. B.Chir. [55], Prof. David Montani, M.D., Ph.D. [10], Prof. Steven D. Nathan, M.D. [56], Michael Newnham, M.B.B.S. [3], Prof. Andrea Olschewski, M.D. [48], Prof. Horst Olschewski, M.D. [48,49], Prof. Ronald J. Oudiz, M.D. [57], Prof. Willem H. Ouwehand, M.D., Ph.D. [4,5], Prof. Andrew J. Peacock, M.D. [28], Joanna Pepke-Zaba, Ph.D. [58], Zia Rehman, M.D. [59], Prof. Ivan M. Robbins, M.D. [60], Prof. Dan M. Roden, M.D. [61,62], Prof. Erika B. Rosenzweig, M.D. [63], Ghulam Saydain, M.D. [64], Laura Scelsi, M.D. [33], Robert Schilz, D.O. [65], Prof. Werner Seeger, M.D. [13], Christian M. Shaffer, M.Sc. [61], Robert W. Simms, M.D. [66], Marc Simon, M.D. [67], Prof. Olivier Sitbon, M.D., Ph.D. [10], Jay Suntharalingam, M.D. [53], Haiyang Tang, Ph.D. [41,80], Alexander Y. Tchourbanov, Ph.D. [68], Thenappan Thenappan, M.D. [69], Prof. Fernando Torres, M.D. [70], Mark R. Toshner, M.D. [3], Carmen M. Treacy, B.Sc. [3,58], Prof. Anton Vonk Noordegraaf, M.D. [25], Quinten Waisfisz, Ph.D. [25], Anna K. Walsworth, B.S. [9], Robert E Walter, M.D. [71], John Wharton, Ph.D. [1], Prof. R. James White, M.D., Ph.D. [72], Jeffrey Wilt, M.D. [73], Stephen J. Wort, Ph.D. [74,7], Delphine Yung, M.D. [75], Allan Lawrie, Ph.D. [76], Prof. Marc Humbert, M.D., Ph.D. [10], Prof. Florent Soubrier, M.D., Ph.D. [8], David-Alexandre Trégouët, Ph.D. [8], **Inga Prokopenko, Ph.D.# [1], Prof. Rick Kittles, Ph.D.# [77], Stefan Gräf, Ph.D.# [3,4,5], Prof. William C. Nichols, Ph.D.# [9], Prof. Richard C. Trembath, F.R.C.P.# [7,78], Ankit A. Desai, M.D.#$ [41,50], Prof. Nicholas W. Morrell, M.D.#$ [3,5], Prof. Martin R. Wilkins, M.D.#$ [1]**
on behalf of The NIHR BioResource – Rare Diseases Consortium, UK PAH Cohort Study Consortium and the US PAH Biobank Consortium

*** these authors contributed equally to this work, # these authors jointly supervised this work**
$ corresponding authors

Corresponding authors contact details:
Ankit A. Desai, Indiana University, Indianapolis, IN, United States: ankdesai@iu.edu;
Nicholas W. Morrell, University of Cambridge, Cambridge, United Kingdom: nwm23@cam.ac.uk;
Martin R. Wilkins, Imperial College London, London, United Kingdom: m.wilkins@imperial.ac.uk.

Affiliations

[1] Centre for Pharmacology & Therapeutics, Department of Medicine, Hammersmith Campus, Imperial College London, London, United Kingdom;
[2] Division of Urology, Department of Surgery, The University of Arizona College of Medicine, Tucson, AZ, United States;
[3] Department of Medicine, University of Cambridge, Cambridge, United Kingdom;
[4] Department of Haematology, University of Cambridge, Cambridge, United Kingdom;
[5] NIHR BioResource - Rare Diseases, Cambridge, United Kingdom;
[6] Molecular and Clinical Sciences Research Institute, St George's University of London, London, United Kingdom;
[7] Division of Genetics and Molecular Medicine, King's College London, London, United Kingdom;
[8] Sorbonne Universités, UPMC Univ. Paris 06, Institut National pour la Santé et la Recherche Médicale (INSERM), Unité Mixte de Recherche en Santé (UMR_S) 1166, Team Genomics & Pathophysiology of Cardiovascular Diseases; ICAN Institute for Cardiometabolism and Nutrition, Paris, France;
[9] Division of Human Genetics, Cincinnati Children's Hospital Medical Center, Department of Pediatrics, University of Cincinnati College of Medicine, Cincinnati OH, United States;
[10] Université Paris-Sud, Faculté de Médecine, Université Paris-Saclay; AP-HP, Service de Pneumologie, Centre de référence de l'hypertension pulmonaire, Hôpital Bicêtre, Le Kremlin-Bicêtre; INSERM UMR_S 999, Hôpital Marie Lannelongue, Le Plessis Robinson, Paris, France;
[11] Data Science Institute, Lancaster University, Lancaster, United Kingdom;
[12] Department of Pharmacy Practice & Science, University of Arizona College of Pharmacy, Sarver Heart Center and Center for Applied Genetics and Genomic Medicine (TCAG2M), University of Arizona College of Medicine, Tucson, AZ, United States;

[13] University of Giessen and Marburg Lung Center (UGMLC), member of the German Center for Lung Research (DZL) and of the Excellence Cluster Cardio-Pulmonary System (ECCCPS), Giessen, Germany;
[14] Section of Epigenomics and Disease, Department of Medicine, Hammersmith Campus, Imperial College London, London, United Kingdom;
[15] Division of Cardiovascular Medicine, University of Iowa, Iowa City IA, United States;
[16] Univ. Lille, Inserm, CHU Lille, Institut Pasteur de Lille, U1167 - RID-AGE - Risk Factors and Molecular Determinants of Aging-related Diseases, F-59000, Lille, France;
[17] Queen's University, Kingston ON, Canada;
[18] Medical University of South Carolina, Charleston SC, United States;
[19] Vanderbilt University-Peds, Nasville TN, United States;
[20] University of Colorado Denver, Aurora CO, United States;
[21] Baylor Research Institute, Plano TX, United States;
[22] Medstar Health, Washington D.C., United States;
[23] Allegheny-Singer Research Institute, Pittsburgh PA, United States;
[24] The Ohio State University, Columbus OH, United States;
[25] VU University Medical Center, Amsterdam, The Netherlands;
[26] Mayo Clinic Florida, Jacksonville FL, United States;
[27] Washington University, St. Louis MO, United States;
[28] Golden Jubilee National Hospital, Glasgow, United Kingdom;
[29] Royal Free Hospital, London, United Kingdom;
[30] Sheffield Pulmonary Vascular Disease Unit, Royal Hallamshire Hospital, Sheffield, United Kingdom;
[31] University of Newcastle, Newcastle, United Kingdom;
[32] Department of Molecular Medicine, University of Pavia, Pavia, Italy;
[33] Fondazione IRCCS Policlinico San Matteo, Pavia, Italy;
[34] INSERM UMR_S 1219, Bordeaux Population Health Research Center, University of Bordeaux, France; Department of Neurology, Bordeaux University Hospital, Bordeaux, France;
[35] Department of Medicine at Intermountain Medical Center and the University of Utah, Murray UT, United States;
[36] University of Cincinnati, Cincinnati OH, United States;
[37] Duke University Medical Center, Durham NC, United States;
[38] Institute of Clinical Molecular Biology, University of Kiel, Kiel, Germany;
[39] Mayo Clinic, Rochester MN, United States;
[40] Weill Cornell Medical College and The Houston Methodist Hospital, Houston TX, United States;
[41] Department of Medicine and Arizona Health Sciences Center, University of Arizona, Tucson, AZ, United States;
[42] National Heart & Lung Institute, Imperial College London and National Pulmonary Hypertension Service, Hammersmith Hospital, London, United Kingdom;
[43] CAGE, Cincinnati Children's Hospital Medical Center, Department of Pediatrics, University of Cincinnati College of Medicine, Cincinnati OH, United States;
[44] Tufts Medical Center, Boston MA, United States;
[45] Cincinnati Children's Hospital Medical Center, Cincinnati OH, United States;
[46] Children's Hospital Colorado, University of Colorado Denver, Aurora CO, United States;
[47] Rhode Island Hospital, Providence RI, United States;
[48] Ludwig Boltzmann Institute for Lung Vascular Research, Graz, Austria;
[49] Medical University of Graz, Graz, Austria;
[50] Indiana University, Indianapolis IN, United States;
[51] Department of Internal Medicine 1, University of Kiel, Kiel, Germany;
[52] Institute of Medical and Biomedical Education, St George's University of London, London, United Kingdom;
[53] Royal United Hospitals Bath NHS Foundation Trust, Bath, United Kingdom;
[54] Division of Biomedical Informatics, Cincinnati Children's Hospital Medical Center, Department of Pediatrics, University of Cincinnati College of Medicine, Cincinnati OH, United States;
[55] Great Ormond Street Hospital, London, United Kingdom;
[56] Inova Heart and Vascular Institute, Falls Church VA, United States;
[57] LA Biomedical Research Institute at Harbor-UCLA, Torrance CA, United States;
[58] Papworth Hospital, Papworth, United Kingdom;
[59] East Carolina University, Greenville NC, United States;
[60] Vanderbilt University Medical Center, Nashville TN, United States;
[61] Department of Medicine, Vanderbilt University School of Medicine, Nashville, TN, United States;
[62] Departments of Biomedical Informatics and Pharmacology, Vanderbilt University School of Medicine, Nashville, TN, United States;
[63] Columbia University, New York NY, United States;
[64] Wayne State University, Detroit MI, United States;
[65] University Hospital of Cleveland, Cleveland OH, United States;
[66] Boston University School of Medicine, Boston MA, United States;
[67] University of Pittsburgh, Pittsburgh PA, United States;
[68] Department of Clinical Genomics, Ambry Genetics, Aliso Viejo, CA, United States;
[69] University of Minnesota, Minneapolis MN, United States;
[70] UT Southwestern, Dallas TX, United States;
[71] LSU Health, Shreveport LA, United States;
[72] University of Rochester Medical Center, Rochester NY, United States;
[73] Spectrum Health Hospitals, Grand Rapids MI, United States;
[74] Royal Brompton Hospital, London, United Kingdom;
[75] Seattle Children's Hospital, Seattle WA, United States;
[76] Department of Infection, Immunity & Cardiovascular Disease, University of Sheffield, Sheffield, United Kingdom;
[77] Division of Health Equities, Department of Population Sciences, City of Hope, Duarte, CA, United States;
[78] Department of Clinical Genetics, Guy's Hospital, London, United Kingdom;
[79] US Department of Veterans Affairs Medical Center, Cincinnati, Ohio, USA;
[80] State Key Laboratory of Respiratory Diseases, Guangzhou Institute of Respiratory Health, The First Affiliated Hospital of Guangzhou Medical University, Guangzhou, Guangdong, China. 510182

NIHR BioResource Collaborators

NIHR BioResource – Rare Disease Consortium[1], Julian Adlard[2], Munaza Ahmed[3], Tim Aitman[4,5], Hana Alachkar[6], David Allsup[7], Jeff Almeida-King[8], Philip Ancliff[9], Richard Antrobus[10], Ruth Armstrong[11,12,13], Gavin Arno[14,15], Sofie Ashford[1,16], William Astle[1,16,17], Anthony Attwood[1,16], Chris Babbs[18,19], Tamam Bakchoul[20], Tadbir Bariana[21,22], Julian Barwell[23,24], David Bennett[25], David Bentley[26], Agnieszka Bierzynska[27], Tina Biss[28], Marta Bleda[29], Harm Bogaard[30], Christian Bourne[26], Sara Boyce[31], John Bradley[1], Gerome Breen[32,33], Paul Brennan[34,35], Carole Brewer[36], Matthew Brown[1,16], Michael Browning[37], Rachel Buchan[38,39], Matthew Buckland[40], Teofila Bueser[41,42,43], Siobhan Burns[40], Oliver Burren[29], Paul Calleja[44], Gerald Carr-White[42], Keren Carss[1,16], Ruth Casey[11,12,13], Mark Caulfield[45], John Chambers[46,47], Jennifer Chambers[48,49], Floria Cheng[49], Patrick F Chinnery[1,50,51], Martin Christian[52], Colin Church[53], Naomi Clements Brod[1,16], Gerry Coghlan[40], Elizabeth Colby[27], Trevor Cole[54], Janine Collins[55], Peter Collins[56], Camilla Colombo[26], Robin Condliffe[57], Stuart Cook[38,58,59,60], Terry Cook[61], Nichola Cooper[62], Paul Corris[63,64], Abigail Crisp-Hihn[1,16], Nicola Curry[65], Cesare Danesino[66], Matthew Daniels[67,68], Louise Daugherty[1,16], John Davis[1,16], Sri V V Deevi[1,16], Timothy Dent[68], Eleanor Dewhurst[1,16], Peter Dixon[48], Kate Downes[1,16], Anna Drazyk[69], Elizabeth Drewe[70], Tina Dutt[71], David Edgar[72], Karen Edwards[1,16], William Egner[73], Wendy Erber[74], Marie Erwood[1,16], Maria C Estiu[75], Gillian Evans[76], Dafydd Gareth Evans[77], Tamara Everington[78], Mélanie Eyries[79], Remi Favier[80,81,82], Debra Fletcher[1,16], James Fox[1,16], Amy Frary[1,16], Courtney French[83], Kathleen Freson[84], Mattia Frontini[1,16], Daniel Gale[85], Henning Gall[86], Claire Geoghegan[26], Terry Gerighty[26], Stefano Ghio[87], Hossein-Ardeschir Ghofrani[62,86], Simon Gibbs[38], Kimberley Gilmour[88], Barbara Girerd[89,90,91], Sarah Goddard[92], Keith Gomez[21,22], Pavels Gordins[93], David Gosal[6], Stefan Gräf[1,16,29], Luigi Grassi[1,16], Daniel Greene[1,16,17], Lynn Greenhalgh[94], Andreas Greinacher[95], Paolo Gresele[96], Philip Griffiths[97,98], Sofia Grigoriadou[99], Russell Grocock[26], Detelina Grozeva[11], Scott Hackett[100], Charaka Hadinnapola[29], William Hague[101], Matthias Haimel[1,16,29], Matthew Hall[70], Helen Hanson[94], Kirsty Harkness[102], Andrew Harper[38,67,103], Claire Harris[64], Daniel Hart[55], Ahamad Hassan[104], Grant Hayman[105], Alex Henderson[106], Jonathan Hoffmann[54], Rita Horvath[107,108], Arjan Houweling[30], Luke Howard[38], Fengyuan Hu[1,16], Gavin Hudson[107], Joseph Hughes[26], Aarnoud Huissoon[100], Marc Humbert[89,90,91], Sean Humphray[26], Sarah Hunter[26], Matthew Hurles[109], Louise Izatt[110], Roger James[1,16], Sally Johnson[111], Stephen Jolles[112,113], Jennifer Jolley[1,16], Neringa Jurkute[14,22], Mary Kasanicki[114], Hanadi Kazkaz[115], Rashid Kazmi[31], Peter Kelleher[39], David Kiely[57], Nathalie Kingston[1], Robert Klima[44], Myrto Kostadima[1,16], Gabor Kovacs[116,117], Ania Koziell[118,119], Roman Kreuzhuber[1,16], Taco Kuijpers[120,121], Ajith Kumar[3], Dinakantha Kumararatne[114], Manju Kurian[122,123], Michael Laffan[124,125], Fiona Lalloo[77], Michele Lambert[126,127], Hana Lango Allen[1,16], Allan Lawrie[128], Mark Layton[124], Claire Lentaigne[124,125], Adam Levine[85], Rachel Linger[1,16], Hilary Longhurst[99], Eleni Louka[18,19], Robert MacKenzie Ross[129], Bella Madan[130], Eamonn Maher[11,131], Jesmeen Maimaris[88], Sarah Mangles[132], Rutendo Mapeta[1,16], Kevin Marchbank[64], Stephen Marks[9], Hugh S Markus[69], Hanns-Ulrich Marschall[133], Andrew Marshall[134,135,136], Jennifer Martin[1,16,29], Mary Mathias[137], Emma Matthews[22,138], Heather Maxwell[139], Paul McAlinden[64], Mark McCarthy[19,103,140], Stuart Meacham[1,16], Adam Mead[141], Karyn Megy[1,16], Sarju Mehta[142], Michel Michaelides[14], Carolyn Millar[124,125], Shahin Moledina[9], David Montani[89,90,91], Tony Moore[14,15], Nicholas Morrell[1,29], Monika Mozere[85], MPGN/C3 Glomerulopathy Rare Renal Disease group[143], Keith Muir[144], Andrew Mumford[145,146], Michael Newnham[29], Jennifer O'Sullivan[130], Samya Obaji[56], Steven Okoli[18,19], Andrea Olschewski[116], Horst Olschewski[116,117], Kai Ren Ong[54], Elizabeth Ormondroyd[67,68], Willem Ouwehand[1,16], Sofia Papadia[1,16], Soo-Mi Park[12,13,147], David Parry[5], Joan Paterson[11,12,13], Andrew Peacock[53], John Peden[26], Kathelijne Peerlinck[84], Christopher Penkett[1,16], Joanna Pepke-Zaba[148], Romina Petersen[1,16], Angela Pyle[107], Stuart Rankin[44], Anupama Rao[9], F Lucy Raymond[1,11], Paula Rayner-Matthews[1,16], Christine Rees[26], Augusto Rendon[45], Tara Renton[43], Andrew Rice[149,150], Sylvia Richardson[17], Alex Richter[10], Irene Roberts[18,19,151], Catherine Roughley[76], Noemi Roy[18,19,151], Omid Sadeghi-Alavijeh[85], Moin Saleem[27], Nilesh Samani[152], Alba Sanchis-Juan[1,16], Ravishankar

Sargur[73], Simon Satchell[27], Sinisa Savic[153], Laura Scelsi[87], Sol Schulman[154], Marie Scully[115], Claire Searle[155], Werner Seeger[86], Carrock Sewell[156], Denis Seyres[1,16], Susie Shapiro[65], Olga Sharmardina[1,16], Rakefet Shtoyerman[157], Keith Sibson[137], Lucy Side[3], Ilenia Simeoni[1,16], Michael Simpson[158], Suthesh Sivapalaratnam[55], Anne-Bine Skytte[159], Katherine Smith[45], Kenneth G C Smith[29,160], Katie Snape[161], Florent Soubrier[79], Simon Staines[1,16], Emily Staples[29], Hannah Stark[1,16], Jonathan Stephens[1,16], Kathleen Stirrups[1,16], Sophie Stock[1,16], Jay Suntharalingam[129], Emilia Swietlik[29], R Campbell Tait[162], Kate Talks[28], Rhea Tan[69], James Thaventhiran[29], Andreas Themistocleous[25], Moira Thomas[163], Kate Thomson[67,68], Adrian Thrasher[9], Chantal Thys[84], Marc Tischkowitz[164], Catherine Titterton[1,16], Cheng-Hock Toh[71], Mark Toshner[29], Matthew Traylor[69], Carmen Treacy[29,148], Richard Trembath[41], Salih Tuna[1,16], Wojciech Turek[44], Ernest Turro[1,16,17], Tom Vale[25], Chris Van Geet[84], Natalie Van Zuydam[25], Marta Vazquez-Lopez[49], Julie von Ziegenweidt[1,16], Anton Vonk Noordegraaf[30], Quintin Waisfisz[30], Suellen Walker[9], James Ware[38,39,58], Hugh Watkins[67,68,103], Christopher Watt[1,16], Andrew Webster[14,15], Wei Wei[50], Steven Welch[100], Julie Wessels[92], Sarah Westbury[145,146], John-Paul Westwood[115], John Wharton[62], Deborah Whitehorn[1,16], James Whitworth[11,12,13], Martin R Wilkins[62], Catherine Williamson[48,165], Edwin Wong[98], Nicholas Wood[166,167], Yvette Wood[1,16], Geoff Woods[11,114], Emma Woodward[77], Stephen Wort[39,41], Austen Worth[9], Katherine Yates[1,16,29], Patrick Yong[168], Tim Young[1,16], Ping Yu[1,16], Patrick Yu-Wai-Man[50]

Affiliations

[1]NIHR BioResource, Cambridge University Hospitals NHS Foundation, Cambridge Biomedical Campus, Cambridge, UK. [2]Chapel Allerton Hospital, Leeds Teaching Hospitals NHS Trust, Leeds, UK. [3]North East Thames Regional Genetics Service, Great Ormond Street Hospital for Children NHS Foundation Trust, London, UK. [4]MRC Clinical Sciences Centre, Faculty of Medicine, Imperial College London, London, UK. [5]Institute of Genetics and Molecular Medicine, University of Edinburgh, Edinburgh, UK. [6]Salford Royal NHS Foundation Trust, Salford, UK. [7]Queens Centre for Haematology and Oncology, Castle Hill Hospital, Hull and East Yorkshire NHS Trust, Cottingham, UK. [8]European Molecular Biology Laboratory, European Bioinformatics Institute (EMBL-EBI), Wellcome Genome Campus, Hinxton, Cambridge, UK. [9]Great Ormond Street Hospital for Children NHS Foundation Trust, London, UK. [10]University Hospitals Birmingham NHS Foundation Trust, Birmingham, UK. [11]Department of Medical Genetics, Cambridge Institute for Medical Research, University of Cambridge, Cambridge Biomedical Campus, Cambridge, UK. [12]Cancer Research UK Cambridge Centre, Cambridge Biomedical Campus, Cambridge, UK. [13]NIHR Cambridge Biomedical Research Centre, Cambridge Biomedical Campus, Cambridge, UK. [14]Moorfields Eye Hospital NHS Foundation Trust, London, UK. [15]UCL Institute of Ophthalmology, University College London, London, UK. [16]Department of Haematology, University of Cambridge, Cambridge Biomedical Campus, Cambridge, UK. [17]MRC Biostatistics Unit, Cambridge Institute of Public Health, University of Cambridge, Cambridge, UK. [18]MRC Molecular Haematology Unit, Weatherall Institute of Molecular Medicine, University of Oxford, Oxford, UK. [19]NIHR Oxford Biomedical Research Centre, Oxford University Hospitals Trust, Oxford, UK. [20]Center for Clinical Transfusion Medicine, University Hospital of Tübingen, Tübingen, Germany. [21]The Katharine Dormandy Haemophilia Centre and Thrombosis Unit, Royal Free London NHS Foundation Trust, London, UK. [22]University College London, London, UK. [23]Department of Clinical Genetics, Leicester Royal Infirmary, University Hospitals of Leicester, Leicester, UK. [24]University of Leicester, Leicester, UK. [25]The Nuffield Department of Clinical Neurosciences, University of Oxford, John Radcliffe Hospital, Oxford, UK. [26]Illumina Limited, Chesterford Research Park, Little Chesterford, Nr Saffron Walden, UK. [27]Bristol Renal, University of Bristol, Bristol, UK. [28]Haematology Department, Royal Victoria Infirmary, The Newcastle upon Tyne Hospitals

NHS Foundation Trust, Newcastle upon Tyne, UK. [29]Department of Medicine, School of Clinical Medicine, University of Cambridge, Cambridge Biomedical Campus, Cambridge, UK. [30]Department of Pulmonary Medicine, VU University Medical Centre, Amsterdam, The Netherlands. [31]Southampton General Hospital, University Hospital Southampton NHS Foundation Trust, Southampton, UK. [32]MRC Social, Genetic & Developmental Psychiatry Centre, Institute of Psychiatry, Psychology & Neuroscience, King's College London, London, UK. [33]NIHR Biomedical Research Centre for Mental Health, Maudsley Hospital, London, UK. [34]Newcastle University, Newcastle upon Tyne, UK. [35]Newcastle upon Tyne Hospitals NHS Foundation Trust, Newcastle upon Tyne, UK. [36]Department of Clinical Genetics, Royal Devon & Exeter Hospital, Royal Devon and Exeter NHS Foundation Trust, Exeter, UK. [37]Department of Immunology, Leicester Royal Infirmary, Leicester, UK. [38]National Heart and Lung Institute, Imperial College London, Royal Brompton Hospital, London, UK. [39]Royal Brompton Hospital, Royal Brompton and Harefield NHS Foundation Trust, London, UK. [40]Royal Free London NHS Foundation Trust, London, UK. [41]King's College London, London, UK. [42]Guy's and St Thomas' Hospital, Guy's and St Thomas' NHS Foundation Trust, London, UK. [43]King's College Hospital NHS Foundation Trust, London, UK. [44]High Performance Computing Service, University of Cambridge, Cambridge, UK. [45]Genomics England Ltd, London, UK. [46]Epidemiology and Biostatistics, Imperial College London, London, UK. [47]Imperial College Healthcare NHS Trust, London, UK. [48]Division of Women's Health, King's College London, London, UK. [49]Women's Health Research Centre, Surgery and Cancer, Faculty of Medicine, Hammersmith Hospital, Imperial College Healthcare NHS Trust, London, UK. [50]Department of Clinical Neurosciences, School of Clinical Medicine, University of Cambridge, Cambridge Biomedical Campus, Cambridge, UK. [51]Medical Research Council Mitochondrial Biology Unit, Cambridge Biomedical Campus, Cambridge, UK. [52]Children's Renal and Urology Unit, Nottingham Children's Hospital, QMC, Nottingham University Hospitals NHS Trust, Nottingham, UK. [53]Golden Jubilee National Hospital, Glasgow, UK. [54]West Midlands Regional Genetics Service, Birmingham Women's and Children's NHS Foundation Trust, Birmingham, UK. [55]The Royal London Hospital, Barts Health NHS Foundation Trust, London, UK. [56]The Arthur Bloom Haemophilia Centre, University Hospital of Wales, Cardiff, UK. [57]Sheffield Pulmonary Vascular Disease Unit, Royal Hallamshire Hospital NHS Foundation Trust, Sheffield, UK. [58]MRC London Institute of Medical Sciences, Imperial College London, London, UK. [59]National Heart Research Institute Singapore, National Heart Centre Singapore, Singapore, Singapore. [60]Division of Cardiovascular & Metabolic Disorders, Duke-National University of Singapore, Singapore, Singapore. [61]Imperial College Renal and Transplant Centre, Hammersmith Hospital, Imperial College Healthcare NHS Trust, London, UK. [62]Department of Medicine, Imperial College London, London, UK. [63]National Pulmonary Hypertension Service (Newcastle), The Newcastle upon Tyne Hospitals NHS Foundation Trust, Newcastle upon Tyne, UK. [64]Institute of Cellular Medicine, Faculty of Medical Sciences, Newcastle University, Newcastle upon Tyne, UK. [65]Oxford Haemophilia and Thrombosis Centre, The Churchill Hospital, Oxford University Hospitals NHS Trust, Oxford, UK. [66]Department of Molecular Medicine, General Biology, and Medical Genetics Unit, University of Pavia, Pavia, Italy. [67]Department of Cardiovascular Medicine, Radcliffe Department of Medicine, University of Oxford, Oxford, UK. [68]Oxford University Hospitals NHS Foundation Trust, Oxford, UK. [69]Stroke Research Group, Department of Clinical Neurosciences, University of Cambridge, Cambridge Biomedical Campus, Cambridge, UK. [70]Nottingham University Hospitals NHS Trust, Nottingham, UK. [71]The Roald Dahl Haemostasis and Thrombosis Centre, The Royal Liverpool Hospital, Liverpool, UK. [72]Regional Immunology Service, Kelvin Building, Royal Victoria Hospital, Belfast, UK. [73]Sheffield Teaching Hospitals NHS Foundation Trust, Sheffield, UK. [74]Pathology and Laboratory Medicine, University of Western Australia, Crawley, Australia. [75]Ramón Sardá Mother's and Children's Hospital, Buenos Aires, Argentina. [76]Haemophilia Centre, Kent & Canterbury Hospital, East Kent Hospitals University Foundation Trust, Canterbury, UK. [77]Manchester Centre for Genomic Medicine, Saint Mary's Hospital, Manchester, UK. [78]Salisbury District Hospital, Salisbury NHS Foundation

Trust, Salisbury, UK. [79]Departement de genetique, Hopital Pitie-Salpetriere, Paris, France. [80]Service d'Hématologie Biologique, Hôpital d'enfants Armand Trousseau, Paris, France, Paris, France. [81]Inserm U1170, Villejuif, France. [82]Assistance Publique-Hôpitaux de Paris, Département d'Hématologie, Hôpital Armand Trousseau, Paris, France. [83]Department of Paediatrics, School of Clinical Medicine, University of Cambridge , Cambridge Biomedical Campus, Cambridge, UK. [84]Department of Cardiovascular Sciences, Center for Molecular and Vascular Biology, University of Leuven, Leuven, Belgium. [85]UCL Centre for Nephrology, University College London, London, UK. [86]University of Giessen and Marburg Lung Center (UGMLC), Giessen, Germany. [87]Division of Cardiology, Fondazione IRCCS Policlinico S. Matteo, Pavia, Italy. [88]UCL Great Ormond Street Institute of Child Health, London, UK. [89]Universite Paris-Sud, Le Kremlin-Bicêtre, France. [90]Service de Pneumologie, DHU Thorax Innovation, Hôpital Bicêtre, Le Kremlin-Bicêtre, France. [91]INSERM U999, LabEx LERMIT, Centre Chirurgical Marie Lannelongue, Le Plessis Robinson, France. [92]University Hospitals of North Midlands NHS Trust, Stoke-on-Trent, UK. [93]East Yorkshire Regional Adult Immunology and Allergy Unit, Hull Royal Infirmary, Hull & East Yorkshire Hospitals NHS Trust, Hull, UK. [94]Department of Clinical Genetics, Liverpool Women's NHS Foundation, Liverpool, UK. [95]Institute for Immunology and Transfusion Medicine, University of Greifswald, Greifswald, Germany. [96]Section of Internal and Cardiovascular Medicine, University of Perugia, Perugia, Italy. [97]Mitochondrial Research Group, Institute of Genetic Medicine, Newcastle University, Newcastle upon Tyne, UK. [98]Institute of Genetic Medicine, Newcastle University, Newcastle upon Tyne, UK. [99]Barts Health NHS Foundation Trust, London, UK. [100]Birmingham Heartlands Hospital, Heart of England NHS Foundation Trust, Birmingham, UK. [101]ARCH: Australian Research Centre for Health of Women and Babies, Robinson Research Institute, Discipline of Obstetrics and Gynaecology, The University of Adelaide, Women's and Children's Hospital, Adelaide, Australia. [102]Department of Neurology, Sheffield Teaching Hospitals NHS Foundation Trust, Sheffield, UK. [103]Wellcome Trust Centre for Human Genetics, University of Oxford, Oxford, UK. [104]Department of Neurology, Leeds Teaching Hospital NHS Trust, Leeds, UK. [105]Epsom & St Helier University Hospitals NHS Trust, London, UK. [106]Northern Genetics Service, The Newcastle upon Tyne Hospitals NHS Foundation Trust, International Centre for Life, Newcastle upon Tyne, UK. [107]Wellcome Centre for Mitochondrial Research, Institute of Genetic Medicine, Newcastle University, Newcastle upon Tyne, UK. [108]John Walton Muscular Dystrophy Research Centre, Institute of Genetic Medicine, Newcastle University, Newcastle upon Tyne, UK. [109]Wellcome Trust Sanger Institute, Hinxton, Cambridge, UK. [110]Department of Clinical Genetics, Guy's and St Thomas' NHS Foundation Trust, London, UK. [111]Department of Paediatric Nephrology, Great North Children’s Hospital, Newcastle upon Tyne Hospitals NHS Foundation Trust, Newcastle upon Tyne, UK. [112]University Hospital of Wales, Cardiff, UK. [113]Cardiff & Vale University LHB, Cardiff, UK. [114]Addenbrookes Hospital, Cambridge University Hospitals NHS Foundation Trust, Cambridge, UK. [115]University College London Hospitals NHS Foundation Trust, London, UK. [116]Ludwig Boltzmann Institute for Lung Vascular Research, Graz, Austria. [117]Dept of Internal Medicine, Division of Pulmonology, Medical University of Graz, Graz, Austria. [118]Division of Transplantation Immunology and Mucosal Biology, Department of Experimental Immunobiology, Faculty of Life Sciences and Medicine, King’s College London, London, UK. [119]Department of Paediatric Nephrology, Evelina London Children's Hospital, Guy's & St Thomas' NHS Foundation Trust, London, UK. [120]Department of Pediatric Hematology, Immunology, Rheumatology and Infectious Diseases, Emma Children’s Hospital, Academic Medical Center (AMC), University of Amsterdam, Amsterdam, The Netherlands. [121]Department of Clinical Genetics, Academic Medical Center (AMC), University of Amsterdam, Amsterdam, The Netherlands. [122]Molecular Neurosciences, Developmental Neurosciences, UCL Great Ormond Street Institute of Child Health, London, UK. [123]Department of Neurology, Great Ormond Street Hospital for Children NHS Foundation Trust, London, UK. [124]Department of Haematology, Hammersmith Hospital, Imperial College Healthcare NHS Trust, London, UK. [125]Department of Haematology, Imperial College London, London, UK. [126]Division of Hematology, The

Children's Hospital of Philadelphia, Philadelphia, USA. [127]Department of Pediatrics, Perelman School of Medicine at the University of Pennsylvania, Philadelphia, USA. [128]Department of Infection, Immunity & Cardiovascular Disease, University of Sheffield, Sheffield, UK. [129]Royal United Hospitals Bath NHS Foundation Trust, Bath, UK. [130]Department of Haematology, Guy's and St Thomas' NHS Foundation Trust, London, UK. [131]Cambridge NIHR Biomedical Research Centre, Cambridge Biomedical Campus, Cambridge, UK. [132]Haemophilia, Haemostasis and Thrombosis Centre, Hampshire Hospitals NHS Foundation Trust, Basingstoke, UK. [133]Wallenberg Laboratory, Department of Molecular and Clinical Medicine, Sahlgrenska Academy, University of Gothenburg, Gothenburg, Sweden. [134]Faculty of Medical and Human Sciences, Centre for Endocrinology and Diabetes, Institute of Human Development, University of Manchester, Manchester, UK. [135]Department of Clinical Neurophysiology, Manchester Royal Infirmary, Central Manchester University Hospitals National Health Service Foundation Trust, Manchester Academic Health Science Centre, Manchester, UK. [136]National Institute for Health Research/Wellcome Trust Clinical Research Facility, Manchester, UK. [137]Department of Haematology, Great Ormond Street Hospital for Children NHS Foundation Trust, London, UK. [138]The National Hospital for Neurology and Neurosurgery, University College London Hospitals NHS Foundation Trust, London, UK. [139]Royal Hospital for Children, NHS Greater Glasgow and Clyde, Glasgow, UK. [140]Oxford Centre for Diabetes, Endocrinology and Metabolism, University of Oxford, Churchill Hospital, Oxford, UK. [141]Centre for Haematology, Department of Medicine, Hammersmith Hospital, Imperial College Healthcare NHS Trust, London, UK. [142]Department of Clinical Genetics, Addenbrookes Hospital, Cambridge University Hospitals NHS Foundation Trust, Cambridge, UK. [143]MPGN/C3 Glomerulopathy Rare Renal Disease group, , UK. [144]Institute of Neuroscience and Psychology, University of Glasgow, Glasgow, UK. [145]School of Cellular and Molecular Medicine, University of Bristol, Bristol, UK. [146]University Hospitals Bristol NHS Foundation Trust, Bristol, UK. [147]East Anglian Regional Genetics Service, Cambridge University Hospitals NHS Foundation Trust, Cambridge, UK. [148]Royal Papworth Hospital NHS Foundation Trust, Cambridge, UK. [149]Pain Research, Department of Surgery and Cancer, Faculty of Medicine, Imperial College London, London, UK. [150]Chelsea and Westminster Hospital NHS Foundation Trust, London, UK. [151]Department of Paediatrics, Weatherall Institute of Molecular Medicine, University of Oxford, Oxford, UK. [152]Departments of Cardiovascular Sciences and NIHR Leicester Cardiovascular Biomedical Research Unit, University of Leicester, Leicester, UK. [153]The Leeds Teaching Hospitals NHS Trust, Leeds, UK. [154]Beth Israel Deaconess Medical Centre and Harvard Medical School, Boston, USA. [155]Department of Clinical Genetics, Nottingham University Hospitals NHS Trust, Nottingham, UK. [156]Scunthorpe General Hospital, Northern Lincolnshire and Goole NHS Foundation Trust, Scunthorpe, UK. [157]Clinical Genetics Institute, Kaplan Medical Center, Rehovot, Israel. [158]Genetics and Molecular Medicine, King's College London, London, UK. [159]Aarhus University Hospital, Aarhus, Denmark. [160]Cambridge Institute for Medical Research, University of Cambridge, Cambridge Biomedical Campus, Cambridge, UK. [161]Department of Clinical Genetics, St George's University Hospitals NHS Foundation Trust, London, UK. [162]Glasgow Royal Infirmary, NHS Greater Glasgow and Clyde, Glasgow, UK. [163]Gartnavel General Hospital, NHS Greater Glasgow and Clyde, Glasgow, UK. [164]Addenbrooke's Treatment Centre, Addenbrooke's Hospital, Cambridge University Hospitals NHS Foundation Trust, Cambridge, UK. [165]Institute of Reproductive and Developmental Biology, Surgery and Cancer, Hammersmith Hospital, Imperial College Healthcare NHS Trust, London, UK. [166]Department of Molecular Neuroscience, UCL Institute of Neurology, London, UK. [167]UCL Genetics Institute, London, UK. [168]Frimley Park Hospital, NHS Frimley Health Foundation Trust, Camberley, UK.

US PAH Biobank Consortium

Alison Theuer[72], Anagha Malur[59], Ann Williams[36], Anne Dotson[73], Ashley Warden[18], Brandy Harrington[69], Brenda Vang[69], Caitlin Ziemak[22], Nancy Casanova[41], Elizabeth Caskey[71], Catherine MacDonald[41], Courtney Rowley[18], Daniel Larimore[18], Daniela Brady[63], David Tomer[35], Anne Davis[75], Debra Broach[50], Jane Devereux[63], Ellen Lovato[27], Eric Stratton[66], Erin Turk[50], Esperanza Jackson[21], Gina Paciotti[69], Gretchen Peichel[69], Hellina Birru[22], Holly del Junco[20], Rebecca Ingledue[36], Jackie Bruno[72], Jan Drake[24], Jennifer Marks[50], Jessica Pisarcik[67], Jill Spears[18], Joseph Santiago[24], Jordyn DeMartino[18], Joy Beckmann[57], Julia Palmer[18], Karen Visnaw[44], Karla Kennedy[37], Karlise Lewis[46], Kathleen Miller-Reed[46], Kelly Hannon[20], Kimberly McClain[73], Laura Dillon[69], Lekan Olanipekun[70], Lillian Mendibles[41], Lindsey Hawke[17], Linnea Brody[75], Louise Durst[39], Mary Andrews[65], Melissa Allahua[47], Melissa Stratoberdha[26], Merte Lemma[56], Molly Cope[73], Mya Franzo[73], Natalia Feliz[21], Nicholas Hawkes[41], Tracy Norwood[71], Opal Wilson[71], Amy Palmisciano[47], Peggy Gruhlke[39], Priscilla Correa[23], Randy Blake[57], Reema Karnekar[59], Robyn Do[18], Kristal Rohwer[39], Sara Ahmed[22], Kimberly Schiltz[15], Page Scovel[15], Shannon Cordell[60], Sharon Heuerman[27], Shazzra Ahmad[64], Sylwia Szuberla[20], Tammy Roads[36], Thoeun Iem[39], Traci Mcgaha[67], Tracy Urban[46], Valerie Aston[35], Waleed Ayesh[64], Allison Light[72], Abby Arkon[19], Andrea Tavlarides[26], Audrey Anderson[21], John Bindu[57], Deedre Boekwig[35], Donna Singleton[71], Inna Abrea[26], Jennifer Lee[40], Mark Ormiston[17], Renesa Whitman[21], Royanne Holy[40], Sisama Almeida-Peters[37], Tosin Igenoza[70], Hap Farber[66], Auvo Reponen[45], Mukta Barve[45], Amber Gygi[45], Clayborne Winslow[45]

[9] Division of Human Genetics, Cincinnati Children's Hospital Medical Center, Department of Pediatrics, University of Cincinnati College of Medicine, Cincinnati, OH, United States;
[15] Division of Cardiovascular Medicine, University of Iowa, Iowa City IA, United States;
[17] Queen's University, Kingston ON, Canada;
[18] Medical University of South Carolina, Charleston SC, United States;
[19] Vanderbilt University-Peds, Nashville TN, United States;
[20] University of Colorado Denver, Aurora CO, United States;
[21] Baylor Research Institute, Plano TX, United States;
[22] Medstar Health, Washington D.C., United States;
[23] Allegheny-Singer Research Institute, Pittsburgh PA, United States;
[24] Ohio State University, Columbus OH, United States;
[26] Mayo Clinic Florida, Jacksonville FL, United States;
[27] Washington University, St. Louis MO, United States;
[35] Department of Medicine at Intermountain Medical Center and the University of Utah, Murray UT, United States;
[36] University of Cincinnati, Cincinnati OH, United States;
[37] Duke University Medical Center, Durham NC, United States;
[39] Mayo Clinic, Rochester MN, United States;
[40] Weill Cornell Medical College and The Houston Methodist Hospital, Houston TX, United States;
[41] Department of Medicine and Arizona Health Sciences Center, University of Arizona, Tucson, AZ, United States;
[44] Tufts Medical Center, Boston MA, United States;
[45] Cincinnati Children's Hospital, Cincinnati OH, United States;
[46] Children's Hospital of Colorado, University of Colorado Denver, Aurora CO, United States;
[47] Rhode Island Hospital, Providence RI, United States;
[50] Indiana University, Indianapolis IN, United States;
[56] Inova Heart and Vascular Institute, Falls Church VA, United States;
[57] LA Biomedical Research Institute at Harbor-UCLA, Torrance CA, United States;
[59] East Carolina University, Greenville NC, United States;

[60] Vanderbilt University Medical Center, Nashville TN, United States;
[63] Columbia University, New York NY, United States;
[64] Wayne State University, Detroit MI, United States;
[65] University Hospital of Cleveland, Cleveland OH, United States;
[66] Boston University School of Medicine, Boston MA, United States;
[67] University of Pittsburgh, Pittsburgh PA, United States;
[69] University of Minnesota, Minneapolis MN, United States;
[70] UT Southwestern, Dallas TX, United States;
[71] LSU Health, Shreveport LA, United States;
[72] University of Rochester Medical Center, Rochester NY, United States;
[73] Spectrum Health Hospitals, Grand Rapids MI, United States;
[75] Seattle Children's Hospital, Seattle WA, United States;
